# Array-based sequencing of filaggrin gene for comprehensive detection of disease-associated variants

**DOI:** 10.1016/j.jaci.2017.10.001

**Published:** 2018-02

**Authors:** X.F. Colin C. Wong, Simon L.I.J. Denil, Jia Nee Foo, Huijia Chen, Angeline Su Ling Tay, Rebecca L. Haines, Mark B.Y. Tang, W.H. Irwin McLean, Aileen Sandilands, Frances J.D. Smith, E. Birgitte Lane, Jianjun Liu, John E.A. Common

**Affiliations:** a Institute of Medical Biology, A*STAR, Singapore, Singapore; b Genome Institute of Singapore, A*STAR, Singapore, Singapore; c Lee Kong Chian School of Medicine, Nanyang Technological University, Singapore, Singapore; d National Skin Centre, Singapore, Singapore; e Dermatology and Genetic Medicine, Division of Biological Chemistry and Drug Discovery, School of Life Sciences, University of Dundee, Dundee, United Kingdom

To the Editor:

The filaggrin gene (*FLG*) is essential for skin differentiation and epidermal barrier formation. *FLG* loss-of-function (LoF) variants are associated with ichthyosis vulgaris and the major genetic risk factor for developing atopic dermatitis (AD).^1^, ^2^, ^3^ Genetic stratification of patients with AD according to *FLG* LoF risk is a common practice for both research and clinical studies; however, few studies comprehensively sequence the entire *FLG* coding region. Most studies that include *FLG* genotyping have screened for common predominant LoF variants to report allele frequencies after full Sanger sequencing of a smaller batch of test patient samples or previously published data. This strategy potentially results in underreporting of the genetic contribution especially in ethnicities where *FLG* LoF variants are highly diverse.^4^ Distinct LoF variants have been reported for most ethnicities studied to date. For example, 2 predominant sequence variants (p.R501X and c.2282del4) make up approximately 80% of the mutation burden in northern Europeans,^5^ whereas in East Asian ethnicities, a larger *FLG* LoF mutation spectrum is found with fewer predominating variants.^6^, ^7^ However, routinely Sanger sequencing the entire *FLG* coding region for large cohorts is not always feasible, although desirable as it is essential to correctly stratify patients. To address this, we developed a robust and cost-effective high-throughput PCR-based method for analyzing the entire coding region of *FLG* using Fluidigm microfluidics technology and next-generation sequencing (NGS). We have applied this method to fully resequence cohorts of Chinese, Malay, and Indian patients with AD from the Singaporean population.

We designed and optimized overlapping *FLG*-specific primer assays (containing NGS adapters) to span the entire *FLG* coding region including known intragenic copy number variation (CNV) (see Fig 1, *A*; see Table E1 in this article's Online Repository at www.jacionline.org). A total of 48 overlapping primer assays, with amplicons of maximum 500 base pairs, provided redundancy for sequencing reads across primer-binding sites and 100% coverage of *FLG* exon bases (see Fig 1, *B*). The Fluidigm Access Array 48.48 integrated fluidic circuit (IFC) chip (Fluidigm, San Francisco, Calif) generates 48 amplicons for 48 different DNA samples in parallel, simultaneously thermocycling 2304 PCR reactions at nanoliter volumes (see Fig E1 and this article's Methods section in the Online Repository at www.jacionline.org). Initially 96 DNA samples were assayed in IFC chips before Illumina MiSeq 2x250 bp read mode sequencing (4 samples failed); 14 samples from this batch of 96 were previously Sanger sequenced for the entire *FLG* coding region.^5^, ^7^ The known *FLG* LoF variant profile was then used to validate LoF variant detection with the IFC and NGS method. We identified all *FLG* LoF variants originally identified by Sanger in the 14 samples as well as additional variants in 2 samples (see Table I) and documented LoF variants in the remaining 78 samples that passed quality control testing (see Table E2 in this article's Online Repository at www.jacionline.org). LoF variants were all confirmed by visual inspection using Integrated Genome Viewer before Sanger sequencing. In addition, we determined the *FLG* CNV of repeats 8 and 10 (an important risk factor for AD^8^) in the 92 samples using relative coverage-based metrics (see Fig E2 in this article's Online Repository at www.jacionline.org).

**Fig 1:**
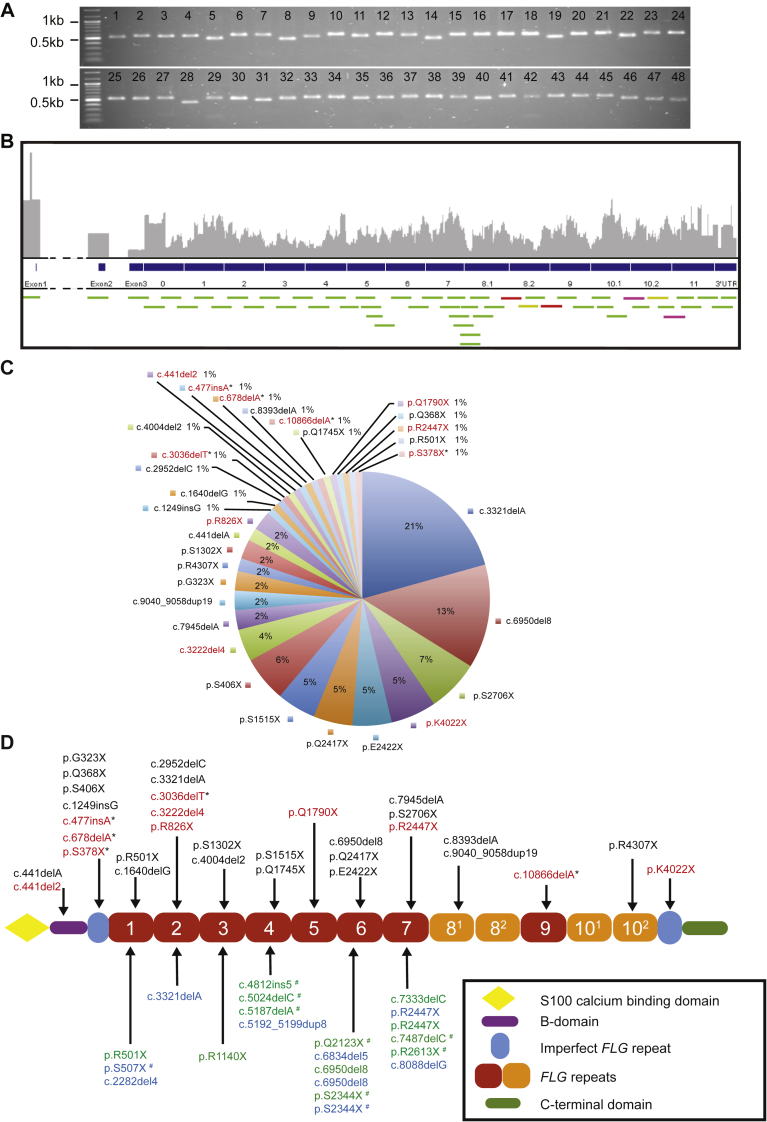
*FLG* primer validation, amplicon coverage, and *FLG* LoF variant analysis in Singaporean cohorts with AD. **A**, A total of 48 *FLG*-specific primer assays were validated by PCR and gel electrophoresis to confirm expected amplicon size and absence of nonspecific products. **B**, Schematic visualization of overlapping amplicon design across 12-repeat *FLG* coding region (green, yellow, red, purple bars). Primer assay 34 (yellow bars), 35 (red bars), and 41 (purple bars) produced multiple distinct amplicons. **C**, Spectrum of disease-associated *FLG* LoF variants identified in the Singaporean Chinese IV and/or AD population; 11 additional variants (highlighted in red) were identified in addition to those from our previous survey, of which 5 variants have not been previously reported (*). **D**, Profilaggrin schematic showing LoF *FLG* variants domain positions—Singaporean Chinese samples are present above the schematic, variants not previously reported in Singapore Chinese samples are highlighted (red) including *FLG* LoF variants not previously published (*). Positions of *FLG* LoF variants identified from Singapore Malays are highlighted in blue and Singapore Indians in green below the schematic. *FLG* LoF variants not previously identified in AD patient-based studies are marked with ^#^. *IV*, Ichthyosis vulgaris.

**Table I:** Concordance of *FLG* LoF variant detection for 14 previously fully Sanger-sequenced AD samples using our MiSeq 2x250 bp protocol

Sample ID	Sanger sequencing	Illumina MiSeq assay
IA-P003	p.S1515X	p.S1515X
IA-P009	No LoF detected	No LoF detected
IA-P014	No LoF detected	No LoF detected
IA-P017	p.E2422X	p.E2422X
IA-P021	p.S406X; c.6950_6957del8	p.S406X; c.6950_6957del8
IA-P024	c.1640delG	c.1640delG
IA-P025	p.Q368X; c.3321delA	p.Q368X; c.3321delA
IA-P028	c.7945delA	c.7945delA
IA-P062	p.Q2417X	p.Q2417X
IA-P063	c.2952delC	c.2952delC
IA-P083∗	c.9040_9058dup19	c.9040_9058dup19; p.Q1790X
IA-P084∗	p.S1302X	p.S1302X; p.S1515X
IA-P090	c.4004del2	c.4004del2
IA-P094	c.2282del4; p.R2447X	c.2282del4; p.R2447X

∗ Our NGS protocol detected additional LoF variants in 2 samples.

The IFC and NGS sequencing method was then used to analyze a further 334 Singaporean ichthyosis vulgaris and/or AD patient samples to obtain estimates of disease-associated LoF allele frequency in the 3 major ethnicities of Singapore—Chinese, Malay, and Indian. In 279 Chinese Singaporean samples (see Table E3 in this article's Online Repository at www.jacionline.org), we identified a further 11 additional LoF variants, raising the total number identified in this population to 33 (an increase from 22 variants identified in our previous study^7^) with 5 not previously reported in the literature (see Fig 1, *C* and *D*); 85 of these samples had also been previously Sanger sequenced^7^ and the concordance profile was near identical (see Table E4 in this article's Online Repository at www.jacionline.org). A total of 14 LoF variants reached significance using Fisher exact test (*P* < .05) compared with population control data derived from ExAC (version 0.3.1) exome database (see Table E5 and the Methods section in this article's Online Repository at www.jacionline.org). The combined *FLG* LoF mutation allele frequency for Chinese Singaporean patients with AD is now 32.3%, an increase from 20.2% in our previous survey of 425 patient samples further supporting the biological importance of *FLG* mutations in AD.^7^ Smaller cohorts of 19 Indian and 36 Malay patients were analyzed and we identified *FLG* LoF variants in 9 Indian samples and 9 Malay samples (see Fig 1, *D*; see Tables E6 and E7 in this article's Online Repository at www.jacionline.org). The identification of unreported *FLG* LoF variants in Indian and Malay ethnicities from Singapore confirms the diversity of *FLG* variants in AD between different ethnic groups. In total we identified 18 variants with limited overlap with the Chinese samples (4 out of 18) and a number of these are not present in the ExAC database, highlighting the contribution of rare, family-specific mutations in AD (11 of 18). This small but well-characterized study of Indian and Malay Singaporeans highlights the variation in combined allele frequencies between ethnicities, with 47.4% of Indian patients with AD and 25% of Malay patients with AD harboring *FLG* LoF variants. The presence of *FLG* LoF variants was strongly associated with AD; however, it was not significantly associated with increasing severity in the 334 patients analyzed in this study, possibly due to the small number of mild cases analzyed (mild cases in this cohort 5.3%; see Table E8 in this article's Online Repository at www.jacionline.org).

In conclusion, we describe a multiplexed targeted resequencing method to study the *FLG* coding region. We highlight that comprehensive sequencing improves accuracy estimates of genetic contribution from *FLG* deleterious alleles and CNVs in AD. This strategy to study genetic variation does not rely on previous mutation spectrum information from any given population or ethnicity and therefore can identify rare, family-specific, or de novo variants globally. This approach outperforms exome sequencing because of its throughput and ability to analyze known CNVs that are not currently reflected in NCBI RefSeq for *FLG*. Amplicon resequencing is robust, reliable, and unbiased and has the potential for scalable sample preparation for small and large research or clinical studies, increasing accurate genotyping for improved outcomes. We have developed a cost-efficient *FLG* genotyping method (∼10 times cheaper than exome sequencing) for researchers and clinicians studying patients from any ethnicity that is vital to advance a precision medicine approach to AD. This method can facilitate research studies immediately and be developed for clinical genetic diagnostics in the future.

## Supplementary data

Online Repository text

Table E1

Table E2

Table E3

Table E4

Table E5

Table E6

Table E7

Table E8

Table E9

Fig E1

Fig E2

Legends for Figs E1 and E2
