# Supplementary material for: Array-based sequencing of filaggrin gene for comprehensive detection of disease-associated variants
Source: J Allergy Clin Immunol. 2018 Feb;141(2):814–6. doi: 10.1016/j.jaci.2017.10.001 (PMC5792052; doi:10.1016/j.jaci.2017.10.001)
Supplement: Online Repository text [file mmc1.docx]

**Supplemental methods**

**Sample collection**

367 DNA samples were extracted from blood or saliva using standard protocols (Oragene, DNA Genotek Inc.; Nucleon Illustra™ BACC2, GE Healthcare). This included 334 patients diagnosed with IV and/or AD, clinically scored with SCORAD and collected with local DSRB approval in accordance with the Declaration of Helsinki (see Table E9 in the Online Repository) ^1^.

**Access Array 48.48 IFC target-specific primer design**

Primer assays were specifically designed to produce amplicons for Illumina MiSeq 2x250 bp read mode sequencing using Reagent Kit-V2. Universal adapter sequences (CS1/CS2) were attached to 3’ ends of primers for sample barcoding (see Table E1 in the Online Repository).

**PCR-based *FLG* enrichment with the Access Array 48.48 IFC**

PCR amplification of *FLG* was performed according to ‘4-Primer Amplicon Tagging on the 48.48 Access Array IFC’ workflow (Chapter 5 Fluidigm user guide: Access Array System for Illumina Sequencing Systems – PN 100-3770 HI, Fluidigm).

Briefly, for each sample a 5 μL sample mix was prepared containing, (1) 50 ng of DNA sample, (2) 2 μM of unique barcoding primer pair (Illumina), (3) FastStart High Fidelity PCR reagents (Roche) and (4) dNTP mix (Bioline). 48 individual DNA containing sample mixtures were loaded into ‘Sample Inlets’ on the Access Array IFC according to manufacturer’s guidelines (see Figure E1 in the Online Repository). Similarly, 48 *FLG* primer assays were loaded into ‘Assay Inlets’ of the Access Array IFC according to manufacturer’s guidelines. Thermocycling of the IFC was completed on Biomark HD PCR machine (Fluidigm) using cycling parameters stated in Table 5 Access Array user guide (PN 100-3770 HI, Fluidigm).

PCR product pools for each individual DNA sample were harvested and quality checked (Agilent DNA 1000 kit, Agilent). Samples were purified with Agencourt^®^ AMPure^®^ XP Reagent Beads (Beckman Coulter Genomics). Batches containing amplicon pools from 96 or 192 individual DNA samples were then further combined in a final pool and sequenced on the Illumina MiSeq (2 x 250 bp read mode).

**Bioinformatics analysis of NGS data**

Sequencing reads were mapped to a *FLG* reference sequence containing 12 repeats ^2^. BWA-MEM (version 0.7.10-r789) was used to map sequencing reads. Mapped reads were stored as indexed BAM files and processed with GATK toolkit (v3.4-46-gbc02625, Java version 1.7.0_75). The *HaplotypeCaller* module was used to calculate SNVs and indels. Individual gVCF files were jointly processed with *GenotypeGVCFs* module to produce a VCF report for multiple samples (same quality cut-offs) to increase the sensitivity of SNV detection ^3^. VCF files were annotated with SnpEff tool (version 4.2) for SNVs and assess their functional impact. These analysis steps were chained together with Pipeline Pilot 2016 (Biovia, Dassault Systems) for consistent data processing and results storage.

**PCR and Sanger-sequencing validation of *FLG* LoF variants and intragenic CNVs**

*FLG* LoF variants were validated with Sanger sequencing using previously published primers ^2, 4^. *FLG* repeat 8 and 10 regions were PCR amplified using Expand High Fidelity^PLUS^ PCR System (Roche). Long-range PCR primers for repeat 8 were 5’-CCCAGGACAAGCAGGAACT-3’ and 5’- GCTTCATGGTGATGCGACCA-3’ ^2^ and for repeat 10 were 5’-GGGCCCAGGACAAGCAGGAAC-3’ (in-house designed) and 5’- CTGCACTACCATAGCTGCC-3’ ^2^. Cycle conditions for repeat 8 PCR were: 95 °C enzyme activation for 5 min, followed by 36 cycles of denaturation (94 °C for 30 s), annealing (64.7 °C for 30 s) and elongation (72 °C for 4 min), followed by a final elongation at 72 °C for 7 min. Cycle conditions for repeat 10 PCR were: 95 °C enzyme activation for 5 min, followed by 35 cycles of denaturation (94 °C for 30 s), annealing (62 °C for 30 s) and elongation (72 °C for 2 min 30 s), followed by a final elongation at 72 °C for 7 min. Intragenic CNVs were distinguished by long-range PCR product sizing on 0.9% w/v agarose gels.

**Statistical analysis of *FLG***

Allele frequencies were analyzed with Fisher’s exact test using a genotype based model (three categories). Population allele frequency control data was extracted from ExAC Database (version 0.3.1) ^5^ using the East Asia subset for comparison with Singaporean Chinese patients. South Asia and East Asia ExAC subset allele frequency data was provided as control data for the Singapore Indian and Malay patients as it was unclear which was the best genetic match. Fisher’s exact test was not performed in Malay and Indian cohorts to avoid any over interpretation of results with small sample sizes. Analyses were performed in R (v3.3.2) with default settings for the Fisher’s exact test function. The association of *FLG* genotype with AD severity – mild, moderate and severe grouping according to oSCORAD ^1^ – was evaluated both with logistic regression and Fisher’s exact tests in R (v3.3.2).

**Supplemental REFERENCes**

1. Oranje AP, Glazenburg EJ, Wolkerstorfer A, de Waard-van der Spek FB. Practical issues on interpretation of scoring atopic dermatitis: the SCORAD index, objective SCORAD and the three-item severity score. Br J Dermatol 2007; 157:645-8.

2. Sandilands A, Terron-Kwiatkowski A, Hull PR, O&apos;Regan GM, Clayton TH, Watson RM, et al. Comprehensive analysis of the gene encoding filaggrin uncovers prevalent and rare mutations in ichthyosis vulgaris and atopic eczema. Nature genetics 2007; 39:650-4.

3. McKenna A, Hanna M, Banks E, Sivachenko A, Cibulskis K, Kernytsky A, et al. The Genome Analysis Toolkit: a MapReduce framework for analyzing next-generation DNA sequencing data. Genome Res 2010; 20:1297-303.

4. Chen H, Common JEA, Haines RL, Balakrishnan A, Brown SJ, Goh CSM, et al. Wide spectrum of filaggrin-null mutations in atopic dermatitis highlights differences between Singaporean Chinese and European populations. The British journal of dermatology 2011.

5. Lek M, Karczewski KJ, Minikel EV, Samocha KE, Banks E, Fennell T, et al. Analysis of protein-coding genetic variation in 60,706 humans. Nature 2016; 536:285-91.
