# Supplementary material for: Array-based sequencing of filaggrin gene for comprehensive detection of disease-associated variants
Source: J Allergy Clin Immunol. 2018 Feb;141(2):814–6. doi: 10.1016/j.jaci.2017.10.001 (PMC5792052; doi:10.1016/j.jaci.2017.10.001)
Supplement: Table E1 [file mmc2.docx]

**Table E1.** Primer assays with a maximum amplicon length of 500 base pairs used for multiplexed PCR amplification on Access Array IFC prior to Illumina MiSeq 2 x 250 sequencing. Primer assays in red text anneal to multiple regions of *FLG* CNV repeats. Primers assays in blue text have been previously published (Sandilands, et al., 2007). Universal CS1 (forward; ACACTGACGACATGGTTCTACA) or CS2 (reverse; TACGGTAGCAGAGACTTGGTCT) NGS adaptor sequences are synthesized 5’ to the *FLG* target sequence.

| **Assay number** | **Primer name** | **Primer Sequence (5'-3')** | **Amplicon Size (bp)** |  | **Start position (HG19)** | **Primer binding location on *FLG* gene** |
| --- | --- | --- | --- | --- | --- | --- |
|  |  |  |  | ***FLG* target region** |  |  |
| 1 | 1_F | CCTAAACTTCCAGAACCTTTTGCC | 450 | Exon 1 | 152297567 | Intron 3’ to exon 1 |
|  | 1_R | CTGTCAAGCCAAAGTGGGGTTA |  |  | 152298016 | Intron 5’ to exon 1 |
| 2 | 2_F | AAGAGCTCAAAATAACCCTTGCT | 498 | Exon 2 | 152287715 | Intron 3’ to exon 2 |
|  | 2_R | ACACTGAGGTCTGTGAGACTACT |  |  | 152288212 | Intron 5’ to exon 2 |
| 3 | 3_F | AGTCTTTCACTTAGCCTCTTCCT | 500 | RPT 0 | 152286751 | RPT 0 |
|  | 3_R | CTCCCTCTGTGACTTCCCTCTG |  |  | 152287250 | 5’ to exon 3 |
| 4 | 4_F | CATCTCTTGACTGCTCCCAC | 500 | RPT 0 | 152286368 | RPT 0 |
|  | 4_R | ACCTACTCATAGAGAAGAAGAATATGGA |  |  | 152286867 | RPT 0 |
| 5 | 5_F | GTCCAGACCGTTCCCCTGAC | 487 | RPT 0 | 152285972 | RPT 0 |
|  | 5_R | GACAGTGAGGGACACTCAGAAG |  |  | 152286458 | RPT 0 |
| 6 | 6_F | AAGCTTCATGATGACGTGACCC | 498 | RPT 0 & 1 | 152285588 | RPT 1 |
|  | 6_R | AGGGACATTCAGAAAACTCAGACA |  |  | 152286085 | RPT 0 |
| 7 | 7_F | CTGCTGACTGGAGCTGGTG | 464 | RPT 1 | 152285210 | RPT 1 |
|  | 7_R | ATCCAGAAGTGCAAGCAGACAAA |  |  | 152285673 | RPT 1 |
| 8 | 8_F | CTATCTACCGATTGCTCGTGGTG | 483 | RPT 1 & 2 | 152284774 | RPT 2 |
|  | 8_R | AAGTGCAGGAGAAAGACATGGAT |  |  | 152285256 | RPT 1 |
| 9 | 9_F | CCGGCTCTGTCTTCGTGAT | 500 | RPT 2 | 152284381 | RPT 2 |
|  | 9_R | AACTCCAGGCACTCAGCAT |  |  | 152284880 | RPT 2 |
| 10 | 10_F | TCACCTGGTAGATGAAAGACCCT | 496 | RPT 2 | 152283998 | RPT 2 |
|  | 10_R | GAAGACTCTGAGAGGTGGTCTGG |  |  | 152284493 | RPT 2 |
| 11 | 11_F | TAGAGCTGTCAGCCCAAGAGG | 495 | RPT 2 & 3 | 152283620 | RPT 3 |
|  | 11_R | CACAGTCAGTGTCAGGCCAT |  |  | 152284114 | RPT 2 |
| 12 | 12_F | GTGCCCAATGCCTGAGTGT | 477 | RPT 3 | 152283237 | RPT 3 |
|  | 12_R | AGCAGACAAACTCGTAAGGACAA |  |  | 152283713 | RPT 3 |
| 13 | 13_F | TCTACTGATTGCTCGTGGTAGGA | 498 | RPT 3 & 4 | 152282833 | RPT 4 |
|  | 13_R | ATCCCATGAACAGGCAAGATCAA |  |  | 152283330 | RPT 3 |
| 14 | 14_F | CTATCTTCTTGATGGGACCTGGG | 498 | RPT 4 | 152282443 | RPT 4 |
|  | 14_R | AAACAGCTCTAGGCACTCAGCAT |  |  | 152282940 | RPT 4 |
| 15 | 15_F | CTGGTAGAGGAAAGACCCTGAAC | 471 | RPT 4 | 152282058 | RPT 4 |
|  | 15_R | GAGTCGGCTTCCAGAAACCATTA |  |  | 152282528 | RPT 4 |
| 16 | 16_F | TTTCTCATTACGTGTTTGTCTGC | 450 | RPT 4 & 5 | 152281746 | RPT 5 |
|  | 16_R | GAGGGACACTCAGAAGAGTCAG |  |  | 152282195 | RPT 4 |
| 17 | 17_F | TCCATGTCTTTCTCCTGCACTTG | 477 | RPT 5 | 152281344 | RPT 5 |
|  | 17_R | TCCCAGGAAAGGTCTGATGTCT |  |  | 152281820 | RPT 5 |
| 18 | 18_F | TGAGTCTTCTGAATGTCCCTCAC | 483 | RPT 5 | 152281200 | RPT 5 |
|  | 18_R | AGCTCTAGACACTCGCAGGT |  |  | 152281682 | RPT 5 |
| 19 | 19_F | TTTCCCTGTGCTGACACTGA | 400 | RPT 5 | 152281171 | RPT 5 |
|  | 19_R | CTGAGAGGTGGTCTGGGTCT |  |  | 152281570 | RPT 5 |
| 20 | 20_F | TCCAGACCTATCTACCGATTGCT | 485 | RPT 5 & 6 | 152280876 | RPT 6 |
|  | 20_R | CAGGAGAAAGACATGGATCCCAC |  |  | 152281360 | RPT 5 |
| 21 | 21_F | CTCTGTCTTCGTGATGGGACCT | 480 | RPT 6 | 152280494 | RPT 6 |
|  | 21_R | CATCCCAAGAGGGTCAGGACA |  |  | 152280973 | RPT 6 |
| 22 | 22_F | GGCGGACTCAGACTGTTCAT | 487 | RPT 6 | 152280081 | RPT 6/7 junction |
|  | 22_R | AAACCATCATGGATCTGCTCAGG |  |  | 152280567 | RPT 6 |
| 23 | 23_F | CGGCCCGAGAGGAAGC | 500 | RPT 6 & 7 | 152279738 | RPT 7 |
|  | 23_R | AGAAGACTCAGACACACAGTCAG |  |  | 152280237 | RPT 6 |
| 24 | 24_F | CTCTGACTGCAGATGAAGCTTGT | 493 | RPT7 | 152279321 | RPT7 |
|  | 24_R | ACTCGTAACGATGAACAATCAGGA |  |  | 152279813 | RPT7 |
| 25 | 25_F | CTCTTGGTGGCTCTGCTGAT | 428 | RPT7 | 152279196 | RPT7 |
|  | 25_R | CTGAGAGGTGGTCTGGGTCT |  |  | 152279623 | RPT7 |
| 26 | 26_F | TCCATGGGAGGACTCAGACT | 489 | RPT 7 | 152279103 | RPT 7/8.1 junction |
|  | 26_R | CATCATGGATCTGCTCAGGA |  |  | 152279591 | RPT 7 |
| 27 | 27_F | GTGTCCACGAATGGTGTCCTG | 482 | RPT 7 & 8.1 | 152278995 | RPT 8.1 |
|  | 27_R | GTCACACACAGACTTCCTCTGG |  |  | 152279476 | RPT 7 |
| 28 | 28_F | GATGTGGTGTGGCTGTGATG | 411 | RPT 7 & 8.1 | 152278900 | RPT 8.1 |
|  | 28_R | CCGAGGGTACAGTGGTAGTCA |  |  | 152279310 | RPT 7 |
| 29 | 29_F | ACGTGTTGTTCTGCTTGCAC | 483 | RPT 7 & 8.1 | 152278836 | RPT 8.1 |
|  | 29_R | AGTGGACACCGAGGGTACAG |  |  | 152279318 | RPT 7 |
| 30 | 30_F | TTGTCTGCTTGCACTTCTGG | 477 | RPT 7 & 8.1 | 152278841 | RPT 8.1 |
|  | 30_R | AGTGGACACCGAGGGTACAG |  |  | 152279318 | RPT 7 |
| 31 | 31_F | GATGGTTTCTGGAAGCAGACCC | 476 | RPT 8.1 | 152278616 | RPT 8.1 |
|  | 31_R | CAGCACTAGAGGAAGACAAGGAT |  |  | 152279091 | RPT 8.1 |
| 32 | 32_F | CATGACCAGCTCTGCCTTCT | 482 | RPT 8.1 | 152278541 | RPT 8.1 |
|  | 32_R | AGACGGTCAGGACACCATTC |  |  | 152279022 | RPT 8.1 |
| 33 | 33_F | GTGGGCAGTCAGGATCCAGAA | 403 | RPT 8.2 | - | RPT 8.2 |
|  | 33_R | GCCTGTCCACCAGAGGAAGC |  |  | - | RPT 8.2 |
| 34 | 34_F | ACTGACTGTGTGTCTGAGTCTTC | 474 | RPT 8.1/8.2/10.2 | 152278270 | RPT 8.1/8.2/10.2 |
|  | 34_R | AGTCCAGGGACAATCAGAGGG |  |  | 152278743 | RPT 8.1/8.2/10.2 |
| 35 | 35_F | ATTACGTGTTGTTCTGCTTGCAC | 500 | RPT 8.1/8.2/9 | 152278833 | RPT 8.2 & 9 |
|  | 35_R | CTGCAGTCAGAGACAGTGGAC |  |  | 152278360 | RPT 8.1 & 8.2 |
| 36 | 36_F | CCAGAGGAAGTCTCTGCGTGA | 468 | RPT 9 | 152277511 | RPT 9 |
|  | 36_R | TTCGGTAGATAGCTCTGGACACT |  |  | 152277978 | RPT 9 |
| 37 | 37_F | TCTCGTGCCTGCTCGT | 499 | RPT 9 & 10.1 | 152277103 | RPT 10.1 |
|  | 37_R | CCAGGTCCCATCACGAAGAC |  |  | 152277601 | RPT 9 |
| 38 | 38_F | TCAGAGTCTTCTGAGTGTCCC | 461 | RPT 10.1 | 152276704 | RPT 10.1 |
|  | 38_R | CATGGGCGGACCAGGA |  |  | 152277164 | RPT 10.1 |
| 39 | 39_F | TGTCTGGAGCCATCTCTTGA | 400 | RPT 10.1 | 152276632 | RPT 10.1 |
|  | 39_R | GAAGGCAGGGATCCCACT |  |  | 152277031 | RPT 10.1 |
| 40 | 40_F | CTGTCCGTGGGCTGACAC | 491 | RPT 10.1 | 152276310 | RPT 10.1 |
|  | 40_R | CCGGCCAGGGACAATCAG |  |  | 152276800 | RPT 10.1 |
| 41 | 41_F | TCATTACGTGTTTCTCTGCTTGC | 499 | RPT 10.1/10.2/11 | 152275915 | RPT 10.2 & 11 |
|  | 41_R | CAGTCAGAGACAGTGGACACC |  |  | 152276413 | RPT 10.1 & 10.2 |
| 42 | 42_F | GTCCACCCATGGACAGTCTGTG | 430 | RPT10.2 | - | RPT10.2 |
|  | 42_R | GCTAACACTGGATCCCTGGCG |  |  | - | RPT10.2 |
| 43 | 43_F | CAGAGAATTCCTCTGGTGGACAGC | 406 | RPT 10.2 & 11 | 152276149 | RPT 10.2 |
|  | 43_R | CGGGATCCTTGTCTTCCTCCAGTA |  |  | - | RPT 11 |
| 44 | 44_F | ACGATGGTTTCTGGAAGCAG | 411 | RPT 11 | 152275698 | RPT 11 |
|  | 44_R | CAAGAGGGTCAGGACACCAT |  |  | 152276108 | RPT 11 |
| 45 | 45_F | CCACGTGACTGTATTCCTGAGTG | 486 | RPT 11 | 152275519 | RPT 11 |
|  | 45_R | CCATCACAGCCACACCACAT |  |  | 152276004 | RPT 11 |
| 46 | 46_F | GAAAGTGAACTTGCTTCATTCTTCT | 486 | RPT 11  & 3’UTR | 152275119 | 3'UTR |
|  | 46_R | TCAGACTCTAGTACCGCTAAGGA |  |  | 152275604 | RPT 11 |
| 47 | 47_F | GATGTGCTAGCCCTGATGTTGAT | 500 | RPT 11  & 3’UTR | 152274758 | 3' UTR |
|  | 47_R | CCCAGGTTTATGTGGCCATTCTA |  |  | 152275257 | RPT 11 |
| 48 | 48_F | CATCTAATTCTGGCCATGGGGAA | 482 | 3' UTR | 152274569 | Intron 3’ to exon 3 |
|  | 48_R | AGTATTTCATTAGTTTGGTGGTAGCTT |  |  | 152275050 | 3' UTR |
