# Supplementary material for: Array-based sequencing of filaggrin gene for comprehensive detection of disease-associated variants
Source: J Allergy Clin Immunol. 2018 Feb;141(2):814–6. doi: 10.1016/j.jaci.2017.10.001 (PMC5792052; doi:10.1016/j.jaci.2017.10.001)
Supplement: Table E2 [file mmc3.docx]

**Table E2.** *FLG* LoF mutation profile of the remaining 78 samples in the pilot run sequenced with the MiSeq 2x250 bp protocol

| **S/N** | **Sample ID** | ***FLG* mutations identified with Illumina MiSeq 2 x 250 assay** |
| --- | --- | --- |
| 1 | ADD1 NJR | No LoF detected |
| 2 | ADD1 TCF | No LoF detected |
| 3 | AD04 | No LoF detected |
| 4 | AD05 YQY | No LoF detected |
| 5 | AD06 TGF | c.3321delA |
| 6 | AD7 | No LoF detected |
| 7 | AD08 | No LoF detected |
| 8 | AD09 | No LoF detected |
| 9 | AD10 | No LoF detected |
| 10 | C04-CX | No LoF detected |
| 11 | C03-VH | No LoF detected |
| 12 | C01-LRH | No LoF detected |
| 13 | EBL-K001 | No LoF detected |
| 14 | EBL-K002 | No LoF detected |
| 15 | EBL-K003 | No LoF detected |
| 16 | EBL-K004 | c.1249insG |
| 17 | EBL-K005 | No LoF detected |
| 18 | EBL-K009 | No LoF detected |
| 19 | EBL-K010 | No LoF detected |
| 20 | EBL-K011 | No LoF detected |
| 21 | EBL-K013 | No LoF detected |
| 22 | EBL-K015 | No LoF detected |
| 23 | EBL-K024 | No LoF detected |
| 24 | EBL-K025 | No LoF detected |
| 25 | EBL-K026 | No LoF detected |
| 26 | 13-S-K-6 | No LoF detected |
| 27 | 13-S-K-12 | No LoF detected |
| 28 | 13-S-K-13 | No LoF detected |
| 29 | 13-S-K-15 | No LoF detected |
| 30 | 13-S-K-16 | No LoF detected |
| 31 | 13-S-K-17 | p.K4022X |
| 32 | IA-P102 | No LoF detected |
| 33 | IA-P103 | No LoF detected |
| 34 | IA-P104 | No LoF detected |
| 35 | IA-P105 | No LoF detected |
| 36 | IA-P106 | No LoF detected |
| 37 | IA-P107 | No LoF detected |
| 38 | IA-P108 | p.S507X |
| 39 | IA-P109 | c.6834del5 |
| 40 | IA-P110 | c.5024delC |
| 41 | IA-P111 | No LoF detected |
| 42 | IA-P112 | No LoF detected |
| 43 | IA-P113 | No LoF detected |
| 44 | IA-P114 | p.S2706X, p.R826X |
| 45 | IA-P115 | p.R2447X, p.R501X |
| 46 | IA-P116 | c.4812ins5 |
| 47 | IA-P117 | No LoF detected |
| 48 | IA-P119 | No LoF detected |
| 49 | IA-P120 | No LoF detected |
| 50 | IA-P121 | No LoF detected |
| 51 | IA-P123 | No LoF detected |
| 52 | IA-P124 | c.5192_5199dup8 |
| 53 | IA-P126 | c.3321delA, p.R826X |
| 54 | IA-P127 | No LoF detected |
| 55 | IA-P128 | No LoF detected |
| 56 | IA-P129 | No LoF detected |
| 57 | IA-P130 | No LoF detected |
| 58 | IA-P131 | No LoF detected |
| 59 | IA-P133 | p.R2613X |
| 60 | IA-P135 | c.7487delC, c.12856del2 |
| 61 | IA-P136 | p.Q2123X |
| 62 | IA-P137 | No LoF detected |
| 63 | IA-P138 | No LoF detected |
| 64 | JOR-RM | No LoF detected |
| 65 | IA-P140 | No LoF detected |
| 66 | IA-P141 | No LoF detected |
| 67 | IA-P142 | p.S2344X |
| 68 | IA-P143 | c.6950_6957del8 |
| 69 | IA-P144 | No LoF detected |
| 70 | IA-P148 | No LoF detected |
| 71 | IA-P149 | p.E2422X |
| 72 | IA-P150 | c.6950_6957del8 |
| 73 | IA-P151 | p.E2422X |
| 74 | IA-P152 | p.K4022X |
| 75 | IA-P153 | No LoF detected |
| 76 | IA-P155 | No LoF detected |
| 77 | IA-P166 | p.K4022X |
| 78 | Rana. M | No LoF detected |
