# Supplementary material for: Array-based sequencing of filaggrin gene for comprehensive detection of disease-associated variants
Source: J Allergy Clin Immunol. 2018 Feb;141(2):814–6. doi: 10.1016/j.jaci.2017.10.001 (PMC5792052; doi:10.1016/j.jaci.2017.10.001)
Supplement: Table E3 [file mmc4.docx]

| **Table E3.** 279 Chinese AD patient demographics and clinical features. NR = Not recorded in clinic | | | | | | | | |  |  |  |
| --- | --- | --- | --- | --- | --- | --- | --- | --- | --- | --- | --- |
|  |  |  |  |  |  |  |  |  |  |  |  |
| **S/N** | **Sample ID** | **BioSample ID** | **Age (years)** | **Gender** | **IV severity** | **AD Objective SCORAD** | **AD Total SCORAD** | **Onset of atopic eczema (years) 1=under 2, 2=2-4 yrs, 3= 5 or above 4=unknown** | **Asthma** | **Allergic Conjuctivo-Rhinitis** | |
|  |  |  |  |  |  |  |  |  |  | **Recurrent sneezing or runny nose** | **Recurrent watery/ itchy eyes** |
|  |  |  |  |  |  |  |  |  |  |  |  |
|  |  |  |  |  |  |  |  |  |  |  |  |
| 1 | IA-P001 | SAMN06199106 | 36 | F | Moderate | 25.5 | 39.5 | 3 | No | Yes | No |
| 2 | IA-P002 | SAMN06199107 | 17 | M | Severe | 39.5 | 56.5 | 3 | No | Yes | Yes |
| 3 | IA-P003 | SAMN06199108 | 33 | F | Moderate | 36 | 48 | 3 | Yes | Yes | No |
| 4 | IA-P005 | SAMN06199110 | 38 | M | Moderate | 50.5 | 66.5 | 3 | Yes | Yes | No |
| 5 | IA-P006 | SAMN06199111 | 30 | M | Moderate | 47 | 50 | 3 | No | No | Yes |
| 6 | IA-P007 | SAMN06199112 | 25 | F | Mild | 58 | 69 | 1 | No | No | No |
| 7 | IA-P008 | SAMN06199113 | 33 | M | Moderate | 42.5 | 53.5 | 3 | No | Yes | Yes |
| 8 | IA-P009 | SAMN06199114 | 25 | F | No IV | 26.1 | 28.1 | 4 | Yes | Yes | Yes |
| 9 | IA-P010 | SAMN06199115 | 29 | M | Mild | 49 | 58 | 1 | Yes | No | No |
| 10 | IA-P011 | SAMN06199116 | 23 | F | Moderate | 65 | 85 | 1 | No | Yes | Yes |
| 11 | IA-P013 | SAMN06199117 | 29 | M | Mild | 23 | 26 | 3 | No | No | No |
| 12 | IA-P014 | SAMN06199118 | 57 | M | Severe | 46 | 53 | 3 | No | No | No |
| 13 | IA-P015 | SAMN06199119 | 16 | M | No IV | 29 | 37 | 1 | Yes | Yes | Yes |
| 14 | IA-P016 | SAMN06199120 | 31 | F | Moderate | 46 | 53 | 2 | Yes | Yes | Yes |
| 15 | IA-P017 | SAMN06199121 | 33 | M | Moderate | 44.5 | 53.5 | 3 | Yes | No | No |
| 16 | IA-P018 | SAMN06199122 | 22 | M | Mild | 39 | 47 | 3 | No | Yes | No |
| 17 | IA-P020 | SAMN06199124 | 20 | M | Severe | 0 | 0 | 4 | Yes | No | No |
| 18 | IA-P021 | SAMN06199125 | 14 | F | Moderate | 26.5 | 32.5 | 3 | No | No | No |
| 19 | IA-P022 | SAMN06199126 | 43 | M | Mild | 39 | 48 | 3 | No | Yes | No |
| 20 | IA-P023 | SAMN06199127 | 27 | M | Mild | 39 | 49 | 3 | No | Yes | Yes |
| 21 | IA-P024 | SAMN06199128 | 30 | M | Moderate | 50.5 | 61.5 | 2 | No | Yes | No |
| 22 | IA-P025 | SAMN06199129 | 20 | M | Severe | 38.7 | 42.7 | 2 | Yes | Yes | No |
| 23 | IA-P026 | SAMN06199130 | 52 | F | Moderate | 39.5 | 46.5 | 3 | No | Yes | Yes |
| 24 | IA-P027 | SAMN06199131 | 44 | F | Severe | 47.5 | 57.5 | 3 | No | No | No |
| 25 | IA-P028 | SAMN06199132 | 17 | M | Moderate | 57.5 | 61.5 | 3 | Yes | Yes | No |
| 26 | IA-P029 | SAMN06199133 | 21 | M | Moderate | 38.9 | 50.9 | 3 | No | No | No |
| 27 | IA-P030 | SAMN06199134 | 18 | M | No IV | 28.5 | 37.5 | 2 | No | Yes | No |
| 28 | IA-P031 | SAMN06199135 | 21 | M | No IV | 57 | 69 | 2 | No | Yes | Yes |
| 29 | IA-P032 | SAMN06199136 | 28 | M | Moderate | 68.5 | 86.5 | 3 | No | Yes | No |
| 30 | IA-P033 | SAMN06199137 | 30 | M | Moderate | 37 | 50 | 3 | No | Yes | Yes |
| 31 | IA-P034 | SAMN06199138 | 21 | M | Moderate | 39 | 46 | 3 | No | Yes | No |
| 32 | IA-P037 | SAMN06199140 | 60 | F | Severe | 46.6 | 56.6 | 3 | Yes | Yes | Yes |
| 33 | IA-P038 | SAMN06199141 | 25 | F | Mild | 21.4 | 27.4 | 3 | No | Yes | No |
| 34 | IA-P040 | SAMN06199142 | 24 | M | Mild | 62.5 | 72.5 | 2 | No | Yes | No |
| 35 | IA-P041 | SAMN06199143 | 22 | M | Moderate | 68.5 | 80.5 | 3 | No | Yes | Yes |
| 36 | IA-P042 | SAMN06199144 | 27 | F | Mild | 57.5 | 68.5 | 3 | Yes | Yes | No |
| 37 | IA-P043 | SAMN06199145 | 16 | F | Moderate | 24.9 | 30.9 | 2 | No | No | No |
| 38 | IA-P044 | SAMN06199146 | 27 | M | No IV | 41.5 | 51.5 | 3 | No | No | No |
| 39 | IA-P045 | SAMN06199147 | 18 | M | Mild | 21.6 | 36.6 | 2 | Yes | Yes | No |
| 40 | IA-P046 | SAMN06199148 | 41 | F | Mild | 0 | 0 | 4 | No | Yes | No |
| 41 | IA-P047 | SAMN06199149 | 34 | F | Mild | 37 | 45 | 1 | Yes | Yes | Yes |
| 42 | IA-P048 | SAMN06199150 | 37 | M | Moderate | 44 | 50 | 3 | Yes | No | No |
| 43 | IA-P049 | SAMN06199151 | 20 | M | No IV | 65 | 76 | 2 | No | Yes | Yes |
| 44 | IA-P051 | SAMN06199153 | 26 | M | Moderate | 68.5 | 77.5 | 2 | Yes | No | No |
| 45 | IA-P052 | SAMN06199154 | 24 | F | Mild | 54 | 70 | 2 | No | Yes | No |
| 46 | IA-P053 | SAMN06199155 | 36 | M | Mild | 47 | 57 | 3 | Yes | Yes | Yes |
| 47 | IA-P054 | SAMN06199156 | 28 | F | Moderate | 65 | 77 | 3 | No | No | No |
| 48 | IA-P055 | SAMN06199157 | 8 | F | Mild | 24.9 | 29.9 | 3 | No | No | No |
| 49 | IA-P056 | SAMN06199158 | 23 | M | Moderate | 43 | 46 | 3 | No | Yes | No |
| 50 | IA-P057 | SAMN06199159 | 28 | M | No IV | 40.5 | 43.5 | 2 | Yes | No | No |
| 51 | IA-P058 | SAMN06199160 | 31 | M | No IV | 29.6 | 33.6 | 3 | Yes | No | No |
| 52 | IA-P059 | SAMN06199161 | 31 | M | Mild | 38 | 55 | 3 | Yes | Yes | Yes |
| 53 | IA-P060 | SAMN06199162 | 33 | M | No IV | 34.5 | 48.5 | 3 | Yes | No | No |
| 54 | IA-P061 | SAMN06199163 | 22 | M | No IV | 21.4 | 27.4 | 3 | Yes | Yes | No |
| 55 | IA-P062 | SAMN06199164 | 23 | M | No IV | 29.6 | 42.6 | 3 | No | Yes | No |
| 56 | IA-P063 | SAMN06199165 | 20 | M | Moderate | 39 | 56 | 1 | Yes | No | No |
| 57 | IA-P064 | SAMN06199166 | 35 | F | Mild | 24.9 | 32.9 | 2 | No | Yes | Yes |
| 58 | IA-P065 | SAMN06199167 | 28 | M | Mild | 21.2 | 27.2 | 3 | No | Yes | No |
| 59 | IA-P067 | SAMN06199169 | 22 | M | Mild | 37 | 42 | 3 | Yes | Yes | No |
| 60 | IA-P068 | SAMN06199170 | 31 | M | No IV | 47.5 | 54.5 | 3 | No | Yes | No |
| 61 | IA-P069 | SAMN06199171 | 20 | M | No IV | 31 | 40 | 2 | Yes | Yes | Yes |
| 62 | IA-P071 | SAMN06199172 | 22 | M | No IV | 25.1 | 42.1 | 3 | Yes | Yes | No |
| 63 | IA-P072 | SAMN06199173 | 29 | M | Mild | 26.5 | 43.5 | 3 | Yes | Yes | No |
| 64 | IA-P073 | SAMN06199174 | 25 | M | No IV | 37.5 | 43.5 | 3 | No | Yes | No |
| 65 | IA-P074 | SAMN06199175 | 26 | M | Severe | 25 | 36 | 3 | No | No | No |
| 66 | IA-P075 | SAMN06199176 | 33 | F | No IV | 21.6 | 23.6 | 3 | No | No | No |
| 67 | IA-P076 | SAMN06199177 | 20 | F | Moderate | 32.5 | 41.5 | 3 | Yes | No | No |
| 68 | IA-P077 | SAMN06199178 | 21 | M | No IV | 39 | 55 | 3 | Yes | No | No |
| 69 | IA-P078 | SAMN06199179 | 28 | M | No IV | 59.5 | 75.5 | 1 | No | Yes | No |
| 70 | IA-P081 | SAMN06199182 | 14 | F | Mild | 33.5 | 48.5 | 3 | Yes | Yes | No |
| 71 | IA-P082 | SAMN06199183 | 30 | M | No IV | 44.5 | 59.5 | 2 | No | No | Yes |
| 72 | IA-P083 | SAMN06199184 | 21 | M | Severe | 66 | 82 | 3 | No | Yes | Yes |
| 73 | IA-P084 | SAMN06199185 | 22 | M | No IV | 75.5 | 83.5 | 1 | Yes | Yes | No |
| 74 | IA-P085 | SAMN06199186 | 30 | F | No IV | 68 | 84 | 3 | No | Yes | Yes |
| 75 | IA-P086 | SAMN06199187 | 35 | M | No IV | 35.5 | 40.5 | 3 | No | No | No |
| 76 | IA-P087 | SAMN06199188 | 21 | M | No IV | 37 | 45 | 3 | Yes | Yes | Yes |
| 77 | IA-P088 | SAMN06199189 | 20 | M | Severe | 50 | 62 | 3 | Yes | No | No |
| 78 | IA-P089 | SAMN06199190 | 26 | F | Mild | 25.7 | 29.7 | 2 | No | Yes | No |
| 79 | IA-P090 | SAMN06199191 | 23 | F | Mild | 25.5 | 35.5 | 3 | Yes | No | No |
| 80 | IA-P091 | SAMN06199192 | 10 | M | Moderate | 0 | 0 | 4 | No | No | No |
| 81 | IA-P092 | SAMN06199193 | 25 | F | No IV | 31.9 | 38.9 | 3 | No | No | No |
| 82 | IA-P093 | SAMN06199194 | 22 | M | Mild | 59 | 73 | 3 | No | No | No |
| 83 | IA-P096 | SAMN06199197 | 19 | M | Moderate | 44.5 | 46.5 | 2 | No | No | No |
| 84 | IA-P098 | SAMN06199198 | 21 | F | Severe | 0 | 0 | 1 | No | Yes | Yes |
| 85 | IA-P100 | SAMN06199200 | 38 | M | Mild | 53 | 70 | 3 | Yes | No | No |
| 86 | IA-P111 | SAMN06199211 | 38 | F | Moderate | 46 | 51 | 3 | No | No | No |
| 87 | IA-P113 | SAMN06199213 | 17 | M | Moderate | 0 | 0 | 3 | Yes | Yes | No |
| 88 | IA-P114 | SAMN06199214 | 14 | M | Moderate | 0 | 12 | NR | No | Yes | Yes |
| 89 | IA-P126 | SAMN06199224 | 31 | M | NR | 47.5 | 47.5 | NR | NR | NR | NR |
| 90 | IA-P138 | SAMN06199234 | 30 | M | Moderate | 44 | 49 | 3 | Yes | Yes | No |
| 91 | IA-P149 | SAMN06199241 | 24 | M | Severe | 17.7 | 34.7 | 3 | No | No | No |
| 92 | IA-P150 | SAMN06199242 | 27 | F | NR | NR | NR | 3 | No | Yes | No |
| 93 | IA-P152 | SAMN06199243 | 32 | M | Mild | 37 | 43 | 2 | Yes | No | Yes |
| 94 | IA-P153 | SAMN06199244 | 17 | M | No IV | 29 | 36 | 3 | No | No | No |
| 95 | IA-P154 | SAMN06199245 | 22 | F | Mild | 32 | 41 | 1 | No | Yes | Yes |
| 96 | IA-P156 | SAMN06199247 | 15 | M | Moderate | 27 | 31 | 1 | No | Yes | No |
| 97 | IA-P157 | SAMN06199248 | 37 | M | Severe | NR | NR | 1 | No | No | No |
| 98 | IA-P158 | SAMN06199249 | 38 | M | Mild | 47 | 58 | 3 | Yes | No | No |
| 99 | IA-P159 | SAMN06199250 | 37 | F | Mild | 42.5 | 46.5 | 1 | No | No | No |
| 100 | IA-P160 | SAMN06199251 | 16 | M | Moderate | 43 | 56 | 1 | No | No | No |
| 101 | IA-P161 | SAMN06199252 | 23 | F | Moderate | 30 | 35 | 2 | No | No | No |
| 102 | IA-P162 | SAMN06199253 | 21 | M | No IV | 19.5 | 27.5 | 4 | No | Yes | Yes |
| 103 | IA-P163 | SAMN06199254 | 15 | F | Mild | 42.5 | 60.5 | 1 | No | Yes | Yes |
| 104 | IA-P164 | SAMN06199255 | 21 | M | Mild | 16 | 22 | 3 | Yes | Yes | Yes |
| 105 | IA-P166 | SAMN06199257 | 27 | M | Moderate | 39 | 54 | 3 | Yes | No | Yes |
| 106 | IA-P167 | SAMN06199258 | 23 | M | No IV | 60 | 74 | 3 | No | No | Yes |
| 107 | IA-P168 | SAMN06199259 | 31 | F | Moderate | 29 | 43 | 3 | Yes | Yes | Yes |
| 108 | IA-P169 | SAMN06199260 | 18 | M | No IV | 15 | 24 | 3 | Yes | Yes | No |
| 109 | IA-P171 | SAMN06199262 | 22 | M | Mild | 32 | 39 | 2 | No | No | No |
| 110 | IA-P172 | SAMN06199263 | 32 | M | Mild | 37.5 | 49.5 | 3 | No | Yes | Yes |
| 111 | IA-P173 | SAMN06199264 | 22 | M | No IV | 35.5 | 51.5 | 1 | Yes | Yes | Yes |
| 112 | IA-P174 | SAMN06199265 | 50 | M | Moderate | 41 | 56 | 1 | Yes | No | No |
| 113 | IA-P175 | SAMN06199266 | 28 | M | Moderate | 46.5 | 64.5 | 2 | No | Yes | No |
| 114 | IA-P176 | SAMN06199267 | 21 | M | Mild | 54.5 | 68.5 | 2 | No | No | Yes |
| 115 | IA-P177 | SAMN06199268 | 53 | M | Mild | 38 | 44 | 3 | No | Yes | Yes |
| 116 | IA-P178 | SAMN06199269 | 21 | M | No IV | 33 | 53 | 3 | Yes | Yes | No |
| 117 | IA-P179 | SAMN06199270 | 23 | M | Mild | 38 | 51 | 3 | Yes | No | No |
| 118 | IA-P182 | SAMN06199272 | 28 | F | Mild | 16 | 30 | 3 | Yes | Yes | Yes |
| 119 | IA-P184 | SAMN06199274 | 21 | F | Mild | 23 | 26 | 1 | No | No | No |
| 120 | IA-P185 | SAMN06199275 | 28 | M | Moderate | 37.5 | 44.5 | 2 | Yes | Yes | No |
| 121 | IA-P186 | SAMN06199276 | 30 | M | Severe | NR | NR | NR | NR | NR | NR |
| 122 | IA-P187 | SAMN06199277 | 21 | M | Mild | 0 | 0 | 3 | No | NR | NR |
| 123 | IA-P188 | SAMN06199278 | 25 | M | Severe | 27.5 | 31.5 | 3 | No | No | No |
| 124 | IA-P189 | SAMN06199279 | 70 | M | Moderate | 26.5 | 35.5 | 3 | No | No | No |
| 125 | IA-P190 | SAMN06199280 | 27 | M | No IV | 28.5 | 36.5 | 1 | Yes | Yes | Yes |
| 126 | IA-P191 | SAMN06199281 | 22 | M | Moderate | 54 | 69 | 3 | Yes | No | No |
| 127 | IA-P192 | SAMN06199282 | 34 | M | No IV | 30.5 | 37.5 | 3 | No | Yes | No |
| 128 | IA-P193 | SAMN06199283 | 33 | M | No IV | 51.5 | 62.5 | 2 | No | No | No |
| 129 | IA-P194 | SAMN06199284 | 24 | M | No IV | NR | NR | 2 | No | No | No |
| 130 | IA-P195 | SAMN06199285 | 22 | M | Mild | 26.5 | 42.5 | 1 | No | No | No |
| 131 | IA-P196 | SAMN06199286 | 28 | M | Mild | 23 | 39 | 3 | No | Yes | No |
| 132 | IA-P197 | SAMN06199287 | 32 | M | Moderate | 30.5 | 38.5 | 2 | Yes | No | No |
| 133 | IA-P199 | SAMN06199289 | 15 | M | No IV | 26.5 | 38.5 | 2 | No | Yes | No |
| 134 | IA-P201 | SAMN06199291 | 28 | M | No IV | NR | 52.6 | 3 | No | Yes | Yes |
| 135 | P002 | SAMN06199293 | 5 | M | Severe | 39 | 43 | 1 | No | Yes | No |
| 136 | P003 | SAMN06199294 | 9 | M | Severe | 41 | 50 | 1 | No | No | No |
| 137 | P004 | SAMN06199295 | 6 | F | No IV | 51 | 61 | 1 | Yes | Yes | No |
| 138 | P005 | SAMN06199296 | 8 | F | No IV | 23 | 27 | 3 | No | No | No |
| 139 | P006 | SAMN06199297 | 7 | F | No IV | 54.5 | 65.5 | 2 | No | No | No |
| 140 | P007 | SAMN06199298 | 5 | M | Severe | 21.4 | 31.4 | 2 | No | No | No |
| 141 | P008 | SAMN06199299 | 10 | M | Mild | 28.8 | 29.8 | 2 | Yes | Yes | No |
| 142 | P009 | SAMN06199300 | 9 | F | Mild | 11.5 | 14.5 | 2 | No | No | No |
| 143 | P010 | SAMN06199301 | 5 | M | Mild | 21.4 | 24.4 | 2 | No | Yes | Yes |
| 144 | P011 | SAMN06199302 | 15 | M | Moderate | 50 | 52 | 1 | No | Yes | Yes |
| 145 | P013 | SAMN06199303 | 13 | M | NR | 36.4 | 40.4 | 3 | No | Yes | No |
| 146 | P014 | SAMN06199304 | 10 | M | Severe | 70.5 | 82.5 | 1 | No | No | No |
| 147 | P015 | SAMN06199305 | 10 | F | No IV | 11.5 | 16.5 | 3 | No | No | No |
| 148 | P017 | SAMN06199307 | 13 | M | Moderate | 40.1 | 49.1 | 3 | Yes | Yes | No |
| 149 | P018 | SAMN06199308 | 9 | F | Severe | 54.5 | 62.5 | 1 | No | No | No |
| 150 | P019 | SAMN06199309 | 5 | M | Mild | 22 | 27 | 2 | No | No | No |
| 151 | P020 | SAMN06199310 | 12 | F | Moderate | 39.6 | 50.6 | 2 | No | No | No |
| 152 | P021 | SAMN06199311 | 4 | F | Severe | 67 | 74 | 1 | No | No | No |
| 153 | P022 | SAMN06199312 | 14 | M | Moderate | 56.1 | 59.1 | 3 | No | No | No |
| 154 | P023 | SAMN06199313 | 8 | M | Mild | 37.4 | 49.4 | 2 | Yes | No | No |
| 155 | P024 | SAMN06199314 | 5 | M | No IV | 41.5 | 54.5 | 1 | No | No | No |
| 156 | P025 | SAMN06199315 | 12 | M | No IV | 25.5 | 38.5 | 1 | No | Yes | No |
| 157 | P026 | SAMN06199316 | 7 | M | Moderate | 37.5 | 45.5 | 1 | Yes | Yes | Yes |
| 158 | P027 | SAMN06199317 | 5 | M | Moderate | 48.9 | 56.9 | 2 | No | Yes | No |
| 159 | P028 | SAMN06199318 | 5 | M | No IV | 18.5 | 26.5 | 2 | No | No | No |
| 160 | P029 | SAMN06199319 | 7 | F | No IV | 32.5 | 38.5 | 2 | No | No | No |
| 161 | P030 | SAMN06199320 | 11 | M | Mild | 37 | 48 | 2 | Yes | Yes | Yes |
| 162 | P031 | SAMN06199321 | 5 | M | Moderate | 33.5 | 43.5 | 1 | Yes | No | Yes |
| 163 | P032 | SAMN06199322 | 19 | M | No IV | 21.5 | 32.5 | 3 | Yes | No | No |
| 164 | P033 | SAMN06199323 | 4 | M | Severe | 67 | 83 | 1 | No | No | No |
| 165 | P034 | SAMN06199324 | 11 | M | Moderate | 35.5 | 37.5 | 3 | Yes | Yes | No |
| 166 | P035 | SAMN06199325 | 9 | M | Moderate | 34 | 46 | 2 | No | No | No |
| 167 | P036 | SAMN06199326 | 12 | F | No IV | 29.5 | 41.5 | 1 | Yes | Yes | No |
| 168 | P037 | SAMN06199327 | 19 | F | Mild | 10.9 | 14.9 | 3 | No | Yes | No |
| 169 | P038 | SAMN06199328 | 6 | M | Moderate | 26.5 | 34.5 | 3 | Yes | No | No |
| 170 | P039 | SAMN06199329 | 8 | F | NR | 7.4 | 9.4 | 1 | No | Yes | NR |
| 171 | P040 | SAMN06199330 | 9 | F | NR | 17 | 23 | 2 | Yes | No | No |
| 172 | P041 | SAMN06199331 | 10 | M | No IV | 21.4 | 28.4 | 3 | No | Yes | Yes |
| 173 | P042 | SAMN06199332 | 17 | M | No IV | 36 | 48 | 3 | Yes | No | No |
| 174 | P043 | SAMN06199333 | 17 | M | No IV | 69 | 80 | 3 | No | Yes | No |
| 175 | P044 | SAMN06199334 | 11 | F | NR | 23 | 33 | 2 | No | No | No |
| 176 | P045 | SAMN06199335 | 13 | F | No IV | 33.1 | 43.1 | 3 | No | Yes | No |
| 177 | P046 | SAMN06199336 | 4 | M | No IV | 38.5 | 54.5 | 1 | No | Yes | No |
| 178 | P047 | SAMN06199337 | 17 | M | No IV | 50.5 | 66.5 | 1 | No | Yes | No |
| 179 | P048 | SAMN06199338 | 8 | M | No IV | 63 | 73 | 1 | Yes | Yes | No |
| 180 | P049 | SAMN06199339 | 7 | F | Mild | 21.8 | 26.8 | 2 | No | Yes | No |
| 181 | P050 | SAMN06199340 | 4 | M | NR | 32.5 | 45.5 | 1 | No | No | No |
| 182 | P051 | SAMN06199341 | 16 | F | No IV | 26.5 | 34.5 | 3 | No | Yes | Yes |
| 183 | P052 | SAMN06199342 | 9 | M | NR | 52 | 62 | 1 | No | No | No |
| 184 | P053 | SAMN06199343 | 4 | F | NR | 36.5 | 45.5 | 1 | No | Yes | Yes |
| 185 | P054 | SAMN06199344 | 7 | M | No IV | 53 | 60 | 1 | No | No | No |
| 186 | P055 | SAMN06199345 | 20 | M | Severe | 14.4 | 17.4 | 4 | No | Yes | No |
| 187 | P056 | SAMN06199346 | 10 | M | No IV | 60 | 78 | 2 | Yes | Yes | Yes |
| 188 | P057 | SAMN06199347 | 19 | M | No IV | 33.5 | 43.5 | 3 | No | Yes | Yes |
| 189 | P058 | SAMN06199348 | 6 | M | No IV | 49.5 | 58.5 | 2 | No | No | No |
| 190 | P059 | SAMN06199349 | 14 | M | No IV | 43.5 | 48.5 | 3 | No | Yes | No |
| 191 | P060 | SAMN06199350 | 9 | M | Mild | 25.3 | 27.3 | 1 | No | Yes | No |
| 192 | P061 | SAMN06199351 | 5 | M | Mild | 21.6 | 27.6 | 1 | Yes | Yes | Yes |
| 193 | P062 | SAMN06199352 | 5 | M | Mild | 42 | 54 | 2 | No | Yes | No |
| 194 | P063 | SAMN06199353 | 6 | F | Mild | 35.5 | 53.5 | 2 | No | Yes | No |
| 195 | P064 | SAMN06199354 | 19 | M | Severe | 37.5 | 51.5 | 1 | No | Yes | No |
| 196 | P065 | SAMN06199355 | 14 | M | Severe | 59.5 | 69.5 | 3 | No | Yes | No |
| 197 | P066 | SAMN06199356 | 17 | M | Mild | 56 | 66 | 1 | No | No | No |
| 198 | P067 | SAMN06199357 | 10 | F | Mild | 33.1 | 39.1 | 2 | No | Yes | Yes |
| 199 | P068 | SAMN06199358 | 5 | M | No IV | 36 | 46 | 1 | No | Yes | No |
| 200 | P069 | SAMN06199359 | 11 | M | Mild | 32.5 | 42.5 | 3 | No | Yes | Yes |
| 201 | P070 | SAMN06199360 | 15 | M | No IV | 62.5 | 69.5 | 3 | No | Yes | No |
| 202 | P071 | SAMN06199361 | 9 | M | Moderate | 50.5 | 57.5 | 1 | No | Yes | No |
| 203 | P072 | SAMN06199362 | 19 | M | Mild | 50.1 | 66.1 | 3 | No | Yes | No |
| 204 | P073 | SAMN06199363 | 19 | M | No IV | 59.5 | 68.5 | 3 | No | No | No |
| 205 | P074 | SAMN06199364 | 19 | M | Mild | 27 | 35 | 3 | No | Yes | Yes |
| 206 | P075 | SAMN06199365 | 20 | M | No IV | 68 | 84 | 3 | No | No | No |
| 207 | P076 | SAMN06199366 | 20 | M | No IV | 59 | 75 | 4 | No | No | No |
| 208 | P077 | SAMN06199367 | 13 | F | No IV | 38.2 | 46.2 | 3 | No | No | No |
| 209 | P078 | SAMN06199368 | 6 | M | No IV | 33.9 | 47.9 | 2 | No | Yes | No |
| 210 | P079 | SAMN06199369 | 11 | F | NR | 60 | 74 | 1 | No | Yes | No |
| 211 | P080 | SAMN06199370 | 9 | M | Mild | 44.5 | 58.5 | 1 | No | No | No |
| 212 | P081 | SAMN06199371 | 6 | M | No IV | 25.5 | 32.5 | 2 | No | Yes | Yes |
| 213 | P082 | SAMN06199372 | 20 | M | No IV | 33.5 | 43.5 | 3 | No | Yes | No |
| 214 | P083 | SAMN06199373 | 8 | M | No IV | 34.5 | 39.5 | 3 | Yes | No | No |
| 215 | P084 | SAMN06199374 | 19 | M | No IV | 57 | 73 | 1 | Yes | Yes | Yes |
| 216 | P085 | SAMN06199375 | 8 | F | No IV | 46.5 | 53.5 | 2 | No | Yes | No |
| 217 | P086 | SAMN06199376 | 14 | F | Mild | 40.5 | 46.5 | 2 | No | No | No |
| 218 | P087 | SAMN06199377 | 17 | M | No IV | 55.2 | 65.2 | 1 | No | Yes | No |
| 219 | P088 | SAMN06199378 | 3 | F | Mild | 21.2 | 23.2 | 1 | Yes | Yes | No |
| 220 | P089 | SAMN06199379 | 18 | M | No IV | 21.8 | 26.8 | 1 | No | No | No |
| 221 | P090 | SAMN06199380 | 3 | M | Severe | 53 | 69 | 1 | No | No | No |
| 222 | P091 | SAMN06199381 | 7 | F | No IV | 38.9 | 50.9 | 1 | No | Yes | Yes |
| 223 | P092 | SAMN06199382 | 10 | M | NR | 29 | 41 | 1 | Yes | Yes | No |
| 224 | P093 | SAMN06199383 | 10 | M | No IV | 31 | 37 | 3 | No | Yes | Yes |
| 225 | P094 | SAMN06199384 | 3 | M | Mild | 22 | 23 | 1 | No | No | No |
| 226 | P095 | SAMN06199385 | 11 | M | Mild | 49.5 | 57.5 | 1 | Yes | Yes | No |
| 227 | P096 | SAMN06199386 | 2 | M | Mild | 42.5 | 58.5 | 1 | No | No | No |
| 228 | P097 | SAMN06199387 | 5 | M | Mild | 36.1 | 49.1 | 1 | No | Yes | Yes |
| 229 | P098 | SAMN06199388 | 4 | F | Mild | 25.3 | 39.3 | 1 | No | No | No |
| 230 | P099 | SAMN06199389 | 12 | M | Moderate | 62.5 | 68.5 | 4 | No | No | No |
| 231 | P100 | SAMN06199390 | 11 | M | No IV | 21.4 | 30.4 | 2 | No | Yes | No |
| 232 | P101 | SAMN06199391 | 6 | M | No IV | 22 | 42 | 2 | Yes | Yes | Yes |
| 233 | P102 | SAMN06199392 | 6 | M | Mild | 18.5 | 25.5 | 2 | No | No | No |
| 234 | P103 | SAMN06199393 | 13 | M | No IV | 63 | 83 | 3 | Yes | Yes | Yes |
| 235 | P104 | SAMN06199394 | 18 | F | No IV | 23 | 31 | 1 | Yes | Yes | Yes |
| 236 | P105 | SAMN06199395 | 17 | M | No IV | 53 | 69 | 3 | Yes | Yes | Yes |
| 237 | P106 | SAMN06199396 | 15 | M | Severe | 37 | 44 | 1 | No | No | No |
| 238 | P107 | SAMN06199397 | 20 | M | No IV | 46.9 | 62.9 | 4 | No | Yes | No |
| 239 | P108 | SAMN06199398 | 8 | M | Mild | 25.3 | 36.3 | 1 | No | Yes | No |
| 240 | P109 | SAMN06199399 | 13 | M | No IV | 45.6 | 51.6 | 3 | NR | Yes | Yes |
| 241 | P110 | SAMN06199400 | 12 | M | Mild | 44 | 57 | 1 | No | Yes | No |
| 242 | P111 | SAMN06199401 | 15 | M | Severe | 51 | 61 | 1 | No | Yes | No |
| 243 | P112 | SAMN06199402 | 5 | M | Mild | 44 | 58 | 2 | Yes | No | No |
| 244 | P113 | SAMN06199403 | 15 | F | No IV | 22 | 26 | 3 | No | Yes | Yes |
| 245 | P114 | SAMN06199404 | 5 | M | No IV | 35.5 | 43.5 | 2 | No | No | No |
| 246 | P116 | SAMN06199405 | 13 | M | No IV | 25.5 | 34.5 | 2 | Yes | No | No |
| 247 | P117 | SAMN06199406 | 16 | F | Moderate | 30 | 34 | 1 | NR | Yes | No |
| 248 | P118 | SAMN06199407 | 8 | F | No IV | 21.4 | 29.4 | 2 | NR | Yes | No |
| 249 | P119 | SAMN06199408 | 7 | F | No IV | 21.2 | 31.2 | 3 | NR | No | No |
| 250 | P120 | SAMN06199409 | 12 | F | No IV | 20.5 | 34.5 | 1 | Yes | Yes | No |
| 251 | P122 | SAMN06199410 | 13 | M | Moderate | 35.6 | 40.6 | 3 | No | Yes | No |
| 252 | P123 | SAMN06199411 | 4 | F | No IV | 41.3 | 49.3 | 1 | NR | Yes | Yes |
| 253 | P124 | SAMN06199412 | 19 | M | No IV | 21.4 | 35.4 | 3 | Yes | Yes | No |
| 254 | P125 | SAMN06199413 | 9 | M | Moderate | 55.5 | 73.5 | 1 | Yes | Yes | Yes |
| 255 | P126 | SAMN06199414 | 3 | M | Mild | 29.6 | 41.6 | 1 | Yes | No | No |
| 256 | P127 | SAMN06199415 | 5 | F | Mild | 42.5 | 51.5 | 1 | Yes | Yes | No |
| 257 | P129 | SAMN06199416 | 12 | F | NR | 34.1 | 46.1 | 3 | No | Yes | No |
| 258 | P130 | SAMN06199417 | 14 | M | No IV | 40.5 | 52.5 | 1 | No | Yes | No |
| 259 | P131 | SAMN06199418 | 2 | M | Severe | 54.5 | 64.5 | 1 | No | No | No |
| 260 | P132 | SAMN06199419 | 20 | M | Mild | 34 | 41 | 3 | Yes | Yes | No |
| 261 | P133 | SAMN06199420 | 7 | F | No IV | 33.5 | 45.5 | 1 | Yes | Yes | Yes |
| 262 | P134 | SAMN06199421 | 18 | M | Mild | 36 | 47 | 3 | No | Yes | No |
| 263 | P135 | SAMN06199422 | 19 | M | Mild | 37.5 | 39.5 | 3 | No | Yes | No |
| 264 | P136 | SAMN06199423 | 20 | M | No IV | 41 | 50 | 3 | Yes | No | No |
| 265 | P137 | SAMN06199424 | 17 | M | No IV | 40 | 51 | 1 | Yes | No | No |
| 266 | P138 | SAMN06199425 | 2 | M | No IV | 40.6 | 52.6 | 1 | No | No | No |
| 267 | P140 | SAMN06199426 | 6 | M | No IV | 44 | 61 | 3 | No | Yes | Yes |
| 268 | P141 | SAMN06199427 | 6 | M | No IV | 2.8 | 7.8 | 2 | No | Yes | Yes |
| 269 | P142 | SAMN06199428 | 13 | M | Mild | 59 | 68 | 2 | No | Yes | No |
| 270 | P143 | SAMN06199429 | 7 | M | Mild | 40.5 | 56.5 | 2 | No | No | No |
| 271 | P144 | SAMN06199430 | 3 | F | Mild | 36.9 | 49.9 | 2 | No | Yes | No |
| 272 | P146 | SAMN06199431 | 11 | F | No IV | 35.4 | 43.4 | 3 | No | No | No |
| 273 | P147 | SAMN06199432 | 13 | M | No IV | 42.5 | 57.5 | 3 | No | Yes | No |
| 274 | P149 | SAMN06199434 | 12 | M | No IV | 32.3 | 41.3 | 3 | Yes | Yes | No |
| 275 | P150 | SAMN06199435 | 7 | M | No IV | 29.2 | 39.2 | 2 | No | No | No |
| 276 | P151 | SAMN06199436 | 12 | M | No IV | 27.5 | 39.5 | 1 | Yes | No | No |
| 277 | P152 | SAMN06199437 | 8 | F | No IV | 47.1 | 51.1 | 3 | No | Yes | No |
| 278 | P157 | SAMN06199438 | 14 | M | Mild | 41 | 54 | 3 | No | Yes | No |
| 279 | P158 | SAMN06199439 | 17 | F | Mild | 63 | 73 | 1 | No | No | No |
