# Supplementary material for: Array-based sequencing of filaggrin gene for comprehensive detection of disease-associated variants
Source: J Allergy Clin Immunol. 2018 Feb;141(2):814–6. doi: 10.1016/j.jaci.2017.10.001 (PMC5792052; doi:10.1016/j.jaci.2017.10.001)
Supplement: Table E4 [file mmc5.docx]

**Table E4.** 85 of the 279 Singaporean Chinese AD samples were previously Sanger sequence and these samples have now also been sequenced with our microfluidics protocol to check for concordance. One difference was observed and highlighted in red text.

| **S/N** | **Sample ID** | ***FLG* mutation identified from previous Sanger sequencing** | ***FLG* mutation identified from Access Array/MiSeq sequencing** |
| --- | --- | --- | --- |
| 1 | IA-P001 | No LoF variant detected | No LoF variant detected |
| 2 | IA-P002 | p.S1515X, c.8393delA | p.S1515X, c.8393delA |
| 3 | IA-P003 | p.S1515X | p.S1515X |
| 4 | IA-P005 | No LoF variant detected | No LoF variant detected |
| 5 | IA-P006 | No LoF variant detected | No LoF variant detected |
| 6 | IA-P007 | No LoF variant detected | No LoF variant detected |
| 7 | IA-P008 | No LoF variant detected | No LoF variant detected |
| 8 | IA-P009 | No LoF variant detected | No LoF variant detected |
| 9 | IA-P010 | No LoF variant detected | No LoF variant detected |
| 10 | IA-P011 | No LoF variant detected | No LoF variant detected |
| 11 | IA-P013 | No LoF variant detected | No LoF variant detected |
| 12 | IA-P014 | No LoF variant detected | No LoF variant detected |
| 13 | IA-P015 | No LoF variant detected | No LoF variant detected |
| 14 | IA-P016 | No LoF variant detected | No LoF variant detected |
| 15 | IA-P017 | p.E2422X | p.E2422X |
| 16 | IA-P018 | No LoF variant detected | No LoF variant detected |
| 17 | IA-P020 | c.9040_9058dup19 | c.9040_9058dup19 |
| 18 | IA-P021 | c.6950_6957del8, p.S406X | c.6950_6957del8, p.S406X |
| 19 | IA-P022 | No LoF variant detected | No LoF variant detected |
| 20 | IA-P023 | No LoF variant detected | No LoF variant detected |
| 21 | IA-P024 | c.1640delG | c.1640delG |
| 22 | IA-P025 | c.3321delA, p.Q368X | c.3321delA, p.Q368X |
| 23 | IA-P026 | No LoF variant detected | No LoF variant detected |
| 24 | IA-P027 | No LoF variant detected | No LoF variant detected |
| 25 | IA-P028 | c.7945delA | c.7945delA |
| 26 | IA-P029 | No LoF variant detected | No LoF variant detected |
| 27 | IA-P030 | No LoF variant detected | No LoF variant detected |
| 28 | IA-P031 | No LoF variant detected | No LoF variant detected |
| 29 | IA-P032 | No LoF variant detected | No LoF variant detected |
| 30 | IA-P033 | c.3321delA | c.3321delA |
| 31 | IA-P034 | p.R4307X, p.S2706X | p.R4307X, p.S2706X |
| 32 | IA-P037 | c.3321delA | c.3321delA |
| 33 | IA-P038 | No LoF variant detected | No LoF variant detected |
| 34 | IA-P040 | No LoF variant detected | No LoF variant detected |
| 35 | IA-P041 | No LoF variant detected | No LoF variant detected |
| 36 | IA-P042 | No LoF variant detected | No LoF variant detected |
| 37 | IA-P043 | p.S406X, p.Q1745X | p.S406X, p.Q1745X |
| 38 | IA-P044 | No LoF variant detected | No LoF variant detected |
| 39 | IA-P045 | No LoF variant detected | No LoF variant detected |
| 40 | IA-P046 | No LoF variant detected | No LoF variant detected |
| 41 | IA-P047 | No LoF variant detected | No LoF variant detected |
| 42 | IA-P048 | c.3321delA, c.3321delA | c.3321delA, c.3321delA |
| 43 | IA-P049 | No LoF variant detected | No LoF variant detected |
| 44 | IA-P051 | No LoF variant detected | No LoF variant detected |
| 45 | IA-P052 | c.3321delA | c.3321delA |
| 46 | IA-P053 | No LoF variant detected | No LoF variant detected |
| 47 | IA-P054 | No LoF variant detected | No LoF variant detected |
| 48 | IA-P055 | No LoF variant detected | No LoF variant detected |
| 49 | IA-P056 | No LoF variant detected | No LoF variant detected |
| 50 | IA-P057 | No LoF variant detected | No LoF variant detected |
| 51 | IA-P058 | No LoF variant detected | No LoF variant detected |
| 52 | IA-P059 | No LoF variant detected | No LoF variant detected |
| 53 | IA-P060 | c.7945delA | c.7945delA |
| 54 | IA-P061 | No LoF variant detected | No LoF variant detected |
| 55 | IA-P062 | p.Q2417X | p.Q2417X |
| 56 | IA-P063 | c.2952delC | c.2952delC |
| 57 | IA-P064 | p.S2706X | p.S2706X |
| 58 | IA-P065 | No LoF variant detected | No LoF variant detected |
| 59 | IA-P067 | No LoF variant detected | No LoF variant detected |
| 60 | IA-P068 | No LoF variant detected | No LoF variant detected |
| 61 | IA-P069 | No LoF variant detected | No LoF variant detected |
| 62 | IA-P071 | No LoF variant detected | No LoF variant detected |
| 63 | IA-P072 | No LoF variant detected | No LoF variant detected |
| 64 | IA-P073 | No LoF variant detected | No LoF variant detected |
| 65 | IA-P074 | c.3321delA | c.3321delA |
| 66 | IA-P075 | No LoF variant detected | No LoF variant detected |
| 67 | IA-P076 | c.3321delA | c.3321delA |
| 68 | IA-P077 | p.Q2417X | p.Q2417X |
| 69 | IA-P078 | c.9040_9058dup19, p.G323X | c.9040_9058dup19, p.G323X |
| 70 | IA-P081 | No LoF variant detected | No LoF variant detected |
| 71 | IA-P082 | No LoF variant detected | No LoF variant detected |
| 72 | IA-P083 | c.9040_9058dup19 | p.1790X, c.9040_9058dup19 |
| 73 | IA-P084 | p.S1515X, p.S1302X | p.S1515X, p.S1302X |
| 74 | IA-P085 | No LoF variant detected | No LoF variant detected |
| 75 | IA-P086 | c.3321delA | c.3321delA |
| 76 | IA-P087 | No LoF variant detected | No LoF variant detected |
| 77 | IA-P088 | c.3321delA, c.3321delA | c.3321delA, c.3321delA |
| 78 | IA-P089 | c.6950_6957del8, p.S2317X | c.6950_6957del8, p.S2317X |
| 79 | IA-P090 | c.4004del2 | c.4004del2 |
| 80 | IA-P091 | c.3321delA | c.3321delA |
| 81 | IA-P092 | No LoF variant detected | No LoF variant detected |
| 82 | IA-P093 | No LoF variant detected | No LoF variant detected |
| 83 | IA-P096 | c.7249C>T, p.Q2417X | c.7249C>T, p.Q2417X |
| 84 | IA-P098 | p.E2422X, p.R501X | p.E2422X, p.R501X |
| 85 | IA-P100 | No LoF variant detected | No LoF variant detected |
