# Supplementary material for: Array-based sequencing of filaggrin gene for comprehensive detection of disease-associated variants
Source: J Allergy Clin Immunol. 2018 Feb;141(2):814–6. doi: 10.1016/j.jaci.2017.10.001 (PMC5792052; doi:10.1016/j.jaci.2017.10.001)
Supplement: Table E5 [file mmc6.docx]

**Table E5.** Fisher’s exact test of Singaporean Chinese *FLG*-null mutations in the fully sequenced atopic dermatitis (AD) cohort of 279 patients compared with ExAC East Asian control data. False discovery rate is shown (FDR). 14 *FLG* LoF mutations reached individual significance of p<0.05 (**bold**)

| ***FLG* mutation** | **Cohort** | ***FLG* Genotype** | | | | ***P*-value** | **FDR *P*-value** |
| --- | --- | --- | --- | --- | --- | --- | --- |
|  |  | **AA** | **Aa** | **aa** | **Total** |  |  |
| **c.441delA** | AD | 277 | 2 | 0 | 279 | 0.020254 | 0.049855 |
|  | ExAC (East Asia) | 4321 | 2 | 0 | 4323 |  |  |
| c.441del2 | AD | 278 | 1 | 0 | 279 | 0.060573 | 0.084276 |
|  | ExAC (East Asia) | 4327 | 0 | 0 | 4327 |  |  |
| c.477insA | AD | 278 | 1 | 0 | 279 | 0.060573 | 0.084276 |
|  | ExAC (East Asia) | 4327 | 0 | 0 | 4327 |  |  |
| c.678delA | AD | 278 | 1 | 0 | 279 | 0.060573 | 0.084276 |
|  | ExAC (East Asia) | 4327 | 0 | 0 | 4327 |  |  |
| **p.G323X** | AD | 276 | 3 | 0 | 279 | 0.002007 | 0.008028 |
|  | ExAC (East Asia) | 4325 | 2 | 0 | 4327 |  |  |
| p.Q368X | AD | 278 | 1 | 0 | 279 | 0.117490 | 0.134274 |
|  | ExAC (East Asia) | 4326 | 1 | 0 | 4327 |  |  |
| p.S378X | AD | 278 | 1 | 0 | 279 | 0.117490 | 0.134274 |
|  | ExAC (East Asia) | 4326 | 1 | 0 | 4327 |  |  |
| **p.S406X** | AD | 272 | 7 | 0 | 279 | 0.000012 | 0.000094 |
|  | ExAC (East Asia) | 4319 | 8 | 0 | 4327 |  |  |
| c.1249insG | AD | 278 | 1 | 0 | 279 | 0.430429 | 0.430429 |
|  | ExAC (East Asia) | 4319 | 8 | 0 | 4327 |  |  |
| p.R501X | AD | 278 | 1 | 0 | 279 | 0.060573 | 0.084276 |
|  | ExAC (East Asia) | 4327 | 0 | 0 | 4327 |  |  |
| c.1640delG | AD | 278 | 1 | 0 | 279 | 0.170969 | 0.188656 |
|  | ExAC (East Asia) | 4325 | 2 | 0 | 4327 |  |  |
| **p.R826X** | AD | 276 | 3 | 0 | 279 | 0.039975 | 0.084276 |
|  | ExAC (East Asia) | 4317 | 10 | 0 | 4327 |  |  |
| c.2952delC | AD | 278 | 1 | 0 | 279 | 0.060573 | 0.084276 |
|  | ExAC (East Asia) | 4327 | 0 | 0 | 4327 |  |  |
| c.3036delT | AD | 278 | 1 | 0 | 279 | 0.060573 | 0.084276 |
|  | ExAC (East Asia) | 4327 | 0 | 0 | 4327 |  |  |
| **c.3222del4** | AD | 274 | 5 | 0 | 279 | 0.003504 | 0.010638 |
|  | ExAC (East Asia) | 4314 | 13 | 0 | 4327 |  |  |
| **c.3321delA** | AD | 254 | 21 | 2 | 279 | 1.90 x 10^-8^ | 2.03 x 10^-7^ |
|  | ExAC (East Asia) | 4245 | 80 | 2 | 4327 |  |  |
| **p.S1302X** | AD | 276 | 3 | 0 | 279 | 0.000220 | 0.001173 |
|  | ExAC (East Asia) | 4327 | 0 | 0 | 4327 |  |  |
| c.4004del2 | AD | 278 | 1 | 0 | 279 | 0.060573 | 0.084276 |
|  | ExAC (East Asia) | 4327 | 0 | 0 | 4327 |  |  |
| **p.S1515X** | AD | 273 | 6 | 0 | 279 | 0.003112 | 0.010638 |
|  | ExAC (East Asia) | 4308 | 19 | 0 | 4327 |  |  |
| p.Q1745X | AD | 278 | 1 | 0 | 279 | 0.060573 | 0.084276 |
|  | ExAC (East Asia) | 4327 | 0 | 0 | 4327 |  |  |
| p.Q1790X | AD | 278 | 1 | 0 | 279 | 0.354729 | 0.378377 |
|  | ExAC (East Asia) | 4317 | 6 | 0 | 4327 |  |  |
| **c.6950_6957del8** | AD | 264 | 15 | 0 | 279 | 3.78 x 10^-19^ | 1.21 x 10^-17^ |
|  | ExAC (East Asia) | 4327 | 0 | 0 | 4327 |  |  |
| **p.Q2417X** | AD | 273 | 6 | 0 | 279 | 0.000467 | 0.002137 |
|  | ExAC (East Asia) | 4315 | 12 | 0 | 4327 |  |  |
| **p.E2422X** | AD | 273 | 6 | 0 | 279 | 0.010488 | 0.027968 |
|  | ExAC (East Asia) | 4304 | 22 | 1 | 4327 |  |  |
| p.R2447X | AD | 278 | 1 | 0 | 279 | 0.117490 | 0.134274 |
|  | ExAC (East Asia) | 4326 | 1 | 0 | 4327 |  |  |
| c.7945delA | AD | 276 | 3 | 0 | 279 | 0.091426 | 0.121902 |
|  | ExAC (East Asia) | 4312 | 15 | 0 | 4327 |  |  |
| **p.S2706X** | AD | 271 | 8 | 0 | 279 | 1.65 x 10^-10^ | 2.64 x 10^-9^ |
|  | ExAC (East Asia) | 4327 | 0 | 0 | 4327 |  |  |
| c.8393delA | AD | 278 | 1 | 0 | 279 | 0.060573 | 0.084276 |
|  | ExAC (East Asia) | 4327 | 0 | 0 | 4327 |  |  |
| **c.9040_9058dup19** | AD | 276 | 3 | 0 | 279 | 0.000220 | 0.001173 |
|  | ExAC (East Asia) | 4327 | 0 | 0 | 4327 |  |  |
| c.10866delA | AD | 278 | 1 | 0 | 279 | 0.117490 | 0.134274 |
|  | ExAC (East Asia) | 4326 | 1 | 0 | 4327 |  |  |
| **p.R4307X** | AD | 277 | 2 | 0 | 279 | 0.003657 | 0.010638 |
|  | ExAC (East Asia) | 4327 | 0 | 0 | 4327 |  |  |
| p.K4022X | AD | 272 | 7 | 0 | 279 | 0.407330 | 0.420469 |
|  | ExAC (East Asia) | 4162 | 163 | 2 | 4327 |  |  |
