# Supplementary material for: Array-based sequencing of filaggrin gene for comprehensive detection of disease-associated variants
Source: J Allergy Clin Immunol. 2018 Feb;141(2):814–6. doi: 10.1016/j.jaci.2017.10.001 (PMC5792052; doi:10.1016/j.jaci.2017.10.001)
Supplement: Table E6 [file mmc7.docx]

| **Table E6.** 36 Singaporean Malay and 19 Singaporean Indian AD patient demographics and clinical features. NR = Not recorded in clinic | | | | | | | | | | |  |  |
| --- | --- | --- | --- | --- | --- | --- | --- | --- | --- | --- | --- | --- |
|  |  |  |  |  |  |  |  |  |  |  |  |  |
| **S/N** | **Sample ID** | **BioSample ID** | **Age** | **Ethnicity** | **Gender** | **IV severity** | **AD Objective SCORAD** | **AD Total SCORAD** | **Onset of atopic eczema (years) 1=under 2, 2=2-4, 3= 5 or above 4=unknown** | **Asthma** | **Allergic conjuctivo-rhinitis** | |
|  |  |  |  |  |  |  |  |  |  |  | **Recurrent sneezing or runny nose** | **Recurrent watery/ itchy eyes** |
| 1 | IA-P019 | SAMN06199218 | 16 | Malay | Male | Moderate | 52 | 66 | 1 | Yes | No | Yes |
|  |  |  |  |  |  |  |  |  |  |  |  |  |
| 2 | IA-P036 | SAMN06199139 | 45 | Malay | Male | Moderate | 31.7 | 42.7 | 3 | No | No | No |
|  |  |  |  |  |  |  |  |  |  |  |  |  |
| 3 | IA-P066 | SAMN06199168 | 28 | Malay | Female | Moderate | 39.5 | 48.5 | 3 | No | Yes | No |
|  |  |  |  |  |  |  |  |  |  |  |  |  |
| 4 | IA-P079 | SAMN06199180 | 21 | Malay | Male | No IV | 35.5 | 44.5 | 2 | Yes | No | No |
|  |  |  |  |  |  |  |  |  |  |  |  |  |
| 5 | IA-P080 | SAMN06199181 | 24 | Malay | Male | No IV | 44.5 | 61.5 | 2 | Yes | Yes | Yes |
|  |  |  |  |  |  |  |  |  |  |  |  |  |
| 6 | IA-P094 | SAMN06199195 | 21 | Malay | Male | Mild | 18.5 | 32.5 | 3 | No | No | Yes |
|  |  |  |  |  |  |  |  |  |  |  |  |  |
| 7 | IA-P099 | SAMN06199199 | 22 | Malay | Male | Mild | 19.5 | 32.5 | 2 | Yes | No | No |
|  |  |  |  |  |  |  |  |  |  |  |  |  |
| 8 | IA-P102 | SAMN06199202 | 21 | Malay | Male | Moderate | 55 | 74 | 3 | Yes | No | No |
|  |  |  |  |  |  |  |  |  |  |  |  |  |
| 9 | IA-P103 | SAMN06199203 | 24 | Malay | Male | Mild | 48.5 | 61.5 | 1 | Yes | Yes | Yes |
|  |  |  |  |  |  |  |  |  |  |  |  |  |
| 10 | IA-P104 | SAMN06199204 | 7 | Malay | Female | Mild | 25.1 | 30.1 | 1 | No | No | No |
|  |  |  |  |  |  |  |  |  |  |  |  |  |
| 11 | IA-P105 | SAMN06199205 | 36 | Malay | Male | No IV | 65 | 82 | 3 | Yes | Yes | Yes |
|  |  |  |  |  |  |  |  |  |  |  |  |  |
| 12 | IA-P106 | SAMN06199206 | 23 | Malay | Male | Mild | 26.1 | 28.1 | 1 | No | No | No |
|  |  |  |  |  |  |  |  |  |  |  |  |  |
| 13 | IA-P107 | SAMN06199207 | 18 | Malay | Male | No IV | 63 | 77 | 3 | No | No | No |
|  |  |  |  |  |  |  |  |  |  |  |  |  |
| 14 | IA-P108 | SAMN06199208 | 21 | Malay | Male | Mild | 59.5 | 76.5 | 1 | Yes | No | No |
|  |  |  |  |  |  |  |  |  |  |  |  |  |
| 15 | IA-P109 | SAMN06199209 | 19 | Malay | Male | No IV | 57 | 69 | 3 | No | No | No |
|  |  |  |  |  |  |  |  |  |  |  |  |  |
| 16 | IA-P112 | SAMN06199212 | 22 | Malay | Female | No IV | 46.5 | 50.5 | 3 | No | No | No |
|  |  |  |  |  |  |  |  |  |  |  |  |  |
| 17 | IA-P117 | SAMN06199217 | 39 | Malay | Male | Moderate | 47 | 53 | 2 | Yes | No | No |
|  |  |  |  |  |  |  |  |  |  |  |  |  |
| 18 | IA-P124 | SAMN06199222 | 21 | Malay | Male | Mild | 32.5 | 41.5 | 3 | No | Yes | No |
|  |  |  |  |  |  |  |  |  |  |  |  |  |
| 19 | IA-P127 | SAMN06199225 | 20 | Malay | Female | No IV | 44 | 54 | 3 | Yes | No | No |
|  |  |  |  |  |  |  |  |  |  |  |  |  |
| 20 | IA-P128 | SAMN06199226 | 31 | Malay | Female | Mild | 65 | 83 | 3 | Yes | No | No |
|  |  |  |  |  |  |  |  |  |  |  |  |  |
| 21 | IA-P130 | SAMN06199228 | 10 | Malay | Male | Mild | 14.2 | 17.2 | 3 | Yes | Yes | No |
|  |  |  |  |  |  |  |  |  |  |  |  |  |
| 22 | IA-P131 | SAMN06199229 | 21 | Malay | Male | Mild | 57 | 77 | 3 | Yes | Yes | Yes |
|  |  |  |  |  |  |  |  |  |  |  |  |  |
| 23 | IA-P137 | SAMN06199233 | 10 | Malay | Female | Moderate | 11.3 | 28.3 | 3 | No | No | Yes |
|  |  |  |  |  |  |  |  |  |  |  |  |  |
| 24 | IA-P140 | SAMN06199235 | 27 | Malay | Male | No IV | 38 | 45 | 1 | Yes | Yes | No |
|  |  |  |  |  |  |  |  |  |  |  |  |  |
| 25 | IA-P141 | SAMN06199236 | 8 | Malay | Male | Mild | 29.2 | 38.2 | 3 | No | No | No |
|  |  |  |  |  |  |  |  |  |  |  |  |  |
| 26 | IA-P142 | SAMN06199237 | 21 | Malay | Female | Moderate | 33.5 | 40.5 | 3 | Yes | Yes | No |
|  |  |  |  |  |  |  |  |  |  |  |  |  |
| 27 | IA-P143 | SAMN06199238 | 24 | Malay | Male | Mild | 52 | 67 | 3 | No | No | No |
|  |  |  |  |  |  |  |  |  |  |  |  |  |
| 28 | IA-P144 | SAMN06199239 | 22 | Malay | Male | Severe | 60 | 75 | 2 | No | Yes | Yes |
|  |  |  |  |  |  |  |  |  |  |  |  |  |
| 29 | IA-P148 | SAMN06199240 | 21 | Malay | Male | Moderate | 0 | 11 | 2 | No | No | No |
|  |  |  |  |  |  |  |  |  |  |  |  |  |
| 30 | IA-P155 | SAMN06199246 | 33 | Malay | Male | No IV | 49 | 64 | 3 | No | No | No |
|  |  |  |  |  |  |  |  |  |  |  |  |  |
| 31 | IA-P165 | SAMN06199256 | 18 | Malay | Male | Mild | 18.5 | 20.5 | 3 | No | No | No |
|  |  |  |  |  |  |  |  |  |  |  |  |  |
| 32 | IA-P170 | SAMN06199261 | 12 | Malay | Male | No IV | 19.5 | 36.5 | 3 | Yes | Yes | No |
|  |  |  |  |  |  |  |  |  |  |  |  |  |
| 33 | IA-P181 | SAMN06199271 | 26 | Malay | Male | Moderate | 32 | 51 | 2 | No | Yes | Yes |
|  |  |  |  |  |  |  |  |  |  |  |  |  |
| 34 | IA-P198 | SAMN06199288 | 28 | Malay | Male | No IV | 15 | 31 | 3 | Yes | No | No |
|  |  |  |  |  |  |  |  |  |  |  |  |  |
| 35 | IA-P200 | SAMN06199290 | 28 | Malay | Male | Moderate | 33 | 43 | 3 | Yes | Yes | No |
|  |  |  |  |  |  |  |  |  |  |  |  |  |
| 36 | P016 | SAMN06199306 | 18 | Malay | Female | No IV | 60 | 68 | 3 | Yes | Yes | No |
|  |  |  |  |  |  |  |  |  |  |  |  |  |
| 37 | IA-P004 | SAMN06199109 | 54 | Indian | Female | Severe | 42.4 | 55.4 | 3 | Yes | No | No |
|  |  |  |  |  |  |  |  |  |  |  |  |  |
| 38 | IA-P050 | SAMN06199152 | 19 | Indian | Male | Moderate | 36 | 48 | 3 | Yes | Yes | No |
|  |  |  |  |  |  |  |  |  |  |  |  |  |
| 39 | IA-P095 | SAMN06199196 | 8 | Indian | Male | Mild | 54 | 66 | 1 | No | Yes | Yes |
|  |  |  |  |  |  |  |  |  |  |  |  |  |
| 40 | IA-P101 | SAMN06199201 | 39 | Indian | Female | Mild | 24.9 | 36.9 | 3 | Yes | No | No |
|  |  |  |  |  |  |  |  |  |  |  |  |  |
| 41 | IA-P110 | SAMN06199210 | 17 | Indian | Male | Moderate | 48.5 | 54.5 | 2 | Yes | No | No |
|  |  |  |  |  |  |  |  |  |  |  |  |  |
| 42 | IA-P115 | SAMN06199215 | 18 | Indian | Male | Mild | 33.5 | 39.5 | 1 | Yes | Yes | No |
|  |  |  |  |  |  |  |  |  |  |  |  |  |
| 43 | IA-P116 | SAMN06199216 | 26 | Indian | Male | No IV | 38 | 38 | 3 | No | Yes | Yes |
|  |  |  |  |  |  |  |  |  |  |  |  |  |
| 44 | IA-P119 | SAMN06199218 | 8 | Indian | Male | No IV | 55 | 73 | 2 | Yes | Yes | Yes |
|  |  |  |  |  |  |  |  |  |  |  |  |  |
| 45 | IA-P120 | SAMN06199219 | 10 | Indian | Male | No IV | 26.1 | 36.1 | 3 | No | Yes | Yes |
|  |  |  |  |  |  |  |  |  |  |  |  |  |
| 46 | IA-P121 | SAMN06199220 | 34 | Indian | Male | Severe | 52 | 68 | 3 | No | Yes | Yes |
|  |  |  |  |  |  |  |  |  |  |  |  |  |
| 47 | IA-P123 | SAMN06199221 | 23 | Indian | Male | NR | 38 | 48 | 3 | Yes | No | No |
|  |  |  |  |  |  |  |  |  |  |  |  |  |
| 48 | IA-P125 | SAMN06199223 | 60 | Indian | Female | Moderate | 10.7 | 21.7 | 3 | No | No | No |
|  |  |  |  |  |  |  |  |  |  |  |  |  |
| 49 | IA-P129 | SAMN06199227 | 29 | Indian | Male | Moderate | 48 | 58 | 3 | Yes | Yes | Yes |
|  |  |  |  |  |  |  |  |  |  |  |  |  |
| 50 | IA-P133 | SAMN06199230 | 25 | Indian | Female | Mild | 44 | 63 | 3 | No | Yes | No |
|  |  |  |  |  |  |  |  |  |  |  |  |  |
| 51 | IA-P135 | SAMN06199231 | 8 | Indian | Female | Moderate | 50 | 70 | 1 | Yes | Yes | Yes |
|  |  |  |  |  |  |  |  |  |  |  |  |  |
| 52 | IA-P136 | SAMN06199232 | 13 | Indian | Female | Mild | 40.5 | 51.5 | 2 | No | No | No |
|  |  |  |  |  |  |  |  |  |  |  |  |  |
| 53 | IA-P183 | SAMN06199273 | 32 | Indian | Male | NR | 42 | 55 | 3 | Yes | No | No |
|  |  |  |  |  |  |  |  |  |  |  |  |  |
| 54 | P001 | SAMN06199292 | 8 | Indian | Male | Mild | 36 | 41 | 2 | Yes | Yes | No |
|  |  |  |  |  |  |  |  |  |  |  |  |  |
| 55 | P148 | SAMN06199433 | 17 | Indian | Female | Moderate | 28.2 | 38.2 | 1 | No | Yes | Yes |
|  |  |  |  |  |  |  |  |  |  |  |  |  |
