# Supplementary material for: Array-based sequencing of filaggrin gene for comprehensive detection of disease-associated variants
Source: J Allergy Clin Immunol. 2018 Feb;141(2):814–6. doi: 10.1016/j.jaci.2017.10.001 (PMC5792052; doi:10.1016/j.jaci.2017.10.001)
Supplement: Table E7 [file mmc8.docx]

**Table E7. 18 *FLG* LoF variants identified in Singapore Malay and Indian IV and/or AD cohorts using MiSeq 2 x 250 bp protocol.** Singaporean Malay and Indian ethnicities have both unique and recurrent *FLG* LoF variants compared to other published studies (see Table E1). * = not previously reported LoF variant in AD related published literature.

| **ID** | ***FLG* mutation** | **Ethnicity** | **dbSNP ID** | **South Asian ExAC MAF** | **East Asian ExAC MAF** |
| --- | --- | --- | --- | --- | --- |
| 1 | p.R501X | Indian | rs61816761 | 21/16512 | 0/8654 |
| 2 | p.S507X * | Malay | - | Not reported | Not reported |
| 3 | c.2282del4 | Malay | rs558269137 | 122/16510 | 0/8654 |
| 4 | c.3321delA | Malay | rs200519781 | 0/16512 | 82/8654 |
| 5 | p.R1140X | Indian | - | 16/16512 | 0/8652 |
| 6 | c.4812ins5 * | Indian | - | Not reported | Not reported |
| 7 | c.5024delC * | Indian | rs749542190 | Not reported | Not reported |
| 8 | c.5187delA * | Indian | - | Not reported | Not reported |
| 9 | c.5192_5199dup8 | Malay | rs754949514 | Not reported | Not reported |
| 10 | p.Q2123X * | Indian | rs145119684 | 8/16512 | 0/8654 |
| 11 | c.6834del5 | Malay | rs772007167 | Not reported | Not reported |
| 12 | c.6950del8 | Malay; Indian | rs578184315 | Not reported | Not reported |
| 13 | p.S2344X * | Malay, Indian | rs372754256 | Not reported | Not reported |
| 14 | c.7333delC | Indian | - | Not reported | Not reported |
| 15 | p.R2447X | Malay, Indian | rs138726443 | 35/16512 | 1/8652 |
| 16 | c.7487delC * | Indian | rs375277670 | 0/16512 | 5/8650 |
| 17 | p.R2613X * | Indian | rs567795279 | Not reported | Not reported |
| 18 | c.8088delG | Malay | - | Not reported | Not reported |
