# Supplementary material for: Array-based sequencing of filaggrin gene for comprehensive detection of disease-associated variants
Source: J Allergy Clin Immunol. 2018 Feb;141(2):814–6. doi: 10.1016/j.jaci.2017.10.001 (PMC5792052; doi:10.1016/j.jaci.2017.10.001)
Supplement: Table E8 [file mmc9.docx]

**Table E8. Summary of patient demographics and clinical features of the 3 ethnicities studied for *FLG* LoF variants.** SCORAD (SCORing AD) index and objective SCORAD (oSCORAD) are used for clinical phenotyping. Complete metadata is provided in Table E3 and Table E6.

|  | **Chinese** | **Malay** | **Indian** |
| --- | --- | --- | --- |
| Subjects demographics | | | |
| Subjects (*n*) | 279 | 36 | 19 |
| Age, mean (range) in years | 18.5 (2-70) | 22.4 (7-45) | 23.6 (8-60) |
| Male:female (*n*) | 202:77 | 28:8 | 12:7 |
| Ichthyosis vulgaris | | | |
| No ichthyosis vulgaris (*n*) | 103 | 12 | 3 |
| Mild (*n*) | 80 | 13 | 6 |
| Moderate-Severe (*n*) | 84 | 11 | 8 |
| Not recorded (*n*) | 12 | 0 | 2 |
| Number of IV patients with *FLG* LoF (%) | 64 (39.0) | 7 (29.2) | 8 (57.1) |
| Atopic dermatitis | | | |
| Mean Total SCORAD (+/- SD) | 47.2 (± 17.3) | 50.6 (± 19.7) | 50.6 (± 13.9) |
| Mean oSCORAD | 37.6 (± 15.0) | 38.8 (± 17.4) | 39.4 (± 11.4) |
| No. of Mild AD / oSCORAD <15 (%) | 14 (5.1) | 3 (8.3) | 1 (5.3) |
| No. of Moderate AD / oSCORAD 15-40 (%) | 151 (54.9) | 16 (44.4) | 8 (42.1) |
| No. of Severe AD / oSCORAD >40 (%) | 110 (40.0) | 17 (47.2) | 10 (52.6) |
| IV only, AD not reported (*n*) | 4 | 0 | 0 |
| Mean oSCORAD of samples with *FLG* LoF | 37.5 (± 15.3) | 38.9 (± 17.6) | 38.2 (± 8.6) |
| Mean oSCORAD of samples with WT *FLG* | 37.6 (± 15.0) | 38.8 (± 17.4) | 40.0 (± 11.3) |
| AD Age of onset<5 years age (*n*) | 143 | 13 | 8 |
| AD Age of onset>5 years age (*n*) | 124 | 23 | 11 |
| AD Age of onset unknown (*n*) | 12 | 0 | 0 |
