# Supplementary material for: Array-based sequencing of filaggrin gene for comprehensive detection of disease-associated variants
Source: J Allergy Clin Immunol. 2018 Feb;141(2):814–6. doi: 10.1016/j.jaci.2017.10.001 (PMC5792052; doi:10.1016/j.jaci.2017.10.001)
Supplement: Table E9 [file mmc10.docx]

| **Table E9.** Accession numbers and Biosample IDs for all 367 samples used in this study. | | | | | |
| --- | --- | --- | --- | --- | --- |
|  |  |  |  |  |  |
| **Sample_ID** | **Accession number** | **BioSampleID** | **LibraryType** |  |  |
| IA-P021 | SRR5140927 | SAMN06199125 | MiSeq 2x250bp |  |  |
| IA-P025 | SRR5140721 | SAMN06199129 | MiSeq 2x250bp |  |  |
| IA-P062 | SRR5140582 | SAMN06199164 | MiSeq 2x250bp |  |  |
| IA-P063 | SRR5140846 | SAMN06199165 | MiSeq 2x250bp |  |  |
| IA-P083 | SRR5140660 | SAMN06199184 | MiSeq 2x250bp |  |  |
| IA-P102 | SRR5140912 | SAMN06199202 | MiSeq 2x250bp |  |  |
| IA-P103 | SRR5140676 | SAMN06199203 | MiSeq 2x250bp |  |  |
| IA-P104 | SRR5140639 | SAMN06199204 | MiSeq 2x250bp |  |  |
| IA-P105 | SRR5140789 | SAMN06199205 | MiSeq 2x250bp |  |  |
| IA-P107 | SRR5140601 | SAMN06199207 | MiSeq 2x250bp |  |  |
| IA-P108 | SRR5140555 | SAMN06199208 | MiSeq 2x250bp |  |  |
| IA-P111 | SRR5140654 | SAMN06199211 | MiSeq 2x250bp |  |  |
| IA-P113 | SRR5140570 | SAMN06199213 | MiSeq 2x250bp |  |  |
| IA-P114 | SRR5140746 | SAMN06199214 | MiSeq 2x250bp |  |  |
| IA-P116 | SRR5140477 | SAMN06199216 | MiSeq 2x250bp |  |  |
| IA-P117 | SRR5140637 | SAMN06199217 | MiSeq 2x250bp |  |  |
| IA-P119 | SRR5140686 | SAMN06199218 | MiSeq 2x250bp |  |  |
| IA-P123 | SRR5140480 | SAMN06199221 | MiSeq 2x250bp |  |  |
| IA-P125 | SRR5140805 | SAMN06199223 | MiSeq 2x250bp |  |  |
| IA-P128 | SRR5140719 | SAMN06199226 | MiSeq 2x250bp |  |  |
| IA-P130 | SRR5140658 | SAMN06199228 | MiSeq 2x250bp |  |  |
| IA-P137 | SRR5140564 | SAMN06199233 | MiSeq 2x250bp |  |  |
| IA-P138 | SRR5140932 | SAMN06199234 | MiSeq 2x250bp |  |  |
| IA-P140 | SRR5140925 | SAMN06199235 | MiSeq 2x250bp |  |  |
| IA-P142 | SRR5140632 | SAMN06199237 | MiSeq 2x250bp |  |  |
| IA-P150 | SRR5140759 | SAMN06199242 | MiSeq 2x250bp |  |  |
| IA-P151 | SRR5140548 | SAMN06199105 | MiSeq 2x250bp |  |  |
| IA-P152 | SRR5140748 | SAMN06199243 | MiSeq 2x250bp |  |  |
| IA-P153 | SRR5140921 | SAMN06199244 | MiSeq 2x250bp |  |  |
| IA-P154 | SRR5140886 | SAMN06199245 | MiSeq 2x250bp |  |  |
| ADD1 NJR | SRR5140611 | SAMN06199086 | MiSeq 2x250bp |  |  |
| AD04 | SRR5140648 | SAMN06199079 | MiSeq 2x250bp |  |  |
| AD06 TGF | SRR5140663 | SAMN06199081 | MiSeq 2x250bp |  |  |
| AD10 | SRR5140645 | SAMN06199084 | MiSeq 2x250bp |  |  |
| EBL-K001 | SRR5140590 | SAMN06199091 | MiSeq 2x250bp |  |  |
| EBL-K002 | SRR5140549 | SAMN06199092 | MiSeq 2x250bp |  |  |
| EBL-K003 | SRR5140809 | SAMN06199093 | MiSeq 2x250bp |  |  |
| EBL-K004 | SRR5140556 | SAMN06199094 | MiSeq 2x250bp |  |  |
| EBL-K005 | SRR5140839 | SAMN06199095 | MiSeq 2x250bp |  |  |
| EBL-K009 | SRR5140891 | SAMN06199096 | MiSeq 2x250bp |  |  |
| EBL-K010 | SRR5140678 | SAMN06199097 | MiSeq 2x250bp |  |  |
| EBL-K011 | SRR5140613 | SAMN06199098 | MiSeq 2x250bp |  |  |
| EBL-K013 | SRR5140757 | SAMN06199099 | MiSeq 2x250bp |  |  |
| EBL-K015 | SRR5140720 | SAMN06199100 | MiSeq 2x250bp |  |  |
| EBL-K024 | SRR5140741 | SAMN06199101 | MiSeq 2x250bp |  |  |
| EBL-K025 | SRR5140566 | SAMN06199102 | MiSeq 2x250bp |  |  |
| EBL-K026 | SRR5140841 | SAMN06199103 | MiSeq 2x250bp |  |  |
| 13-S-K-6 | SRR5140496 | SAMN06199078 | MiSeq 2x250bp |  |  |
| 13-S-K-12 | SRR5140903 | SAMN06199073 | MiSeq 2x250bp |  |  |
| 13-S-K-15 | SRR5140913 | SAMN06199075 | MiSeq 2x250bp |  |  |
| 13-S-K-17 | SRR5140876 | SAMN06199077 | MiSeq 2x250bp |  |  |
| IA-P003 | SRR5140796 | SAMN06199108 | MiSeq 2x250bp |  |  |
| IA-P009 | SRR5140847 | SAMN06199114 | MiSeq 2x250bp |  |  |
| IA-P014 | SRR5140476 | SAMN06199118 | MiSeq 2x250bp |  |  |
| IA-P017 | SRR5140797 | SAMN06199121 | MiSeq 2x250bp |  |  |
| IA-P024 | SRR5140900 | SAMN06199128 | MiSeq 2x250bp |  |  |
| IA-P028 | SRR5140899 | SAMN06199132 | MiSeq 2x250bp |  |  |
| IA-P084 | SRR5140546 | SAMN06199185 | MiSeq 2x250bp |  |  |
| IA-P090 | SRR5140836 | SAMN06199191 | MiSeq 2x250bp |  |  |
| IA-P094 | SRR5140808 | SAMN06199195 | MiSeq 2x250bp |  |  |
| ADD1 TCF | SRR5140821 | SAMN06199087 | MiSeq 2x250bp |  |  |
| AD05 YQY | SRR5140801 | SAMN06199080 | MiSeq 2x250bp |  |  |
| AD7 | SRR5140907 | SAMN06199085 | MiSeq 2x250bp |  |  |
| AD08 | SRR5140824 | SAMN06199082 | MiSeq 2x250bp |  |  |
| AD09 | SRR5140684 | SAMN06199083 | MiSeq 2x250bp |  |  |
| C04-CX | SRR5140634 | SAMN06199090 | MiSeq 2x250bp |  |  |
| C03-VH | SRR5140861 | SAMN06199089 | MiSeq 2x250bp |  |  |
| C01-LRH | SRR5140878 | SAMN06199088 | MiSeq 2x250bp |  |  |
| 13-S-K-13 | SRR5140754 | SAMN06199074 | MiSeq 2x250bp |  |  |
| 13-S-K-16 | SRR5140591 | SAMN06199076 | MiSeq 2x250bp |  |  |
| IA-P106 | SRR5140860 | SAMN06199206 | MiSeq 2x250bp |  |  |
| IA-P109 | SRR5140872 | SAMN06199209 | MiSeq 2x250bp |  |  |
| IA-P110 | SRR5140504 | SAMN06199210 | MiSeq 2x250bp |  |  |
| IA-P112 | SRR5140893 | SAMN06199212 | MiSeq 2x250bp |  |  |
| IA-P115 | SRR5140857 | SAMN06199215 | MiSeq 2x250bp |  |  |
| IA-P120 | SRR5140606 | SAMN06199219 | MiSeq 2x250bp |  |  |
| IA-P121 | SRR5140535 | SAMN06199220 | MiSeq 2x250bp |  |  |
| IA-P124 | SRR5140829 | SAMN06199222 | MiSeq 2x250bp |  |  |
| IA-P126 | SRR5140705 | SAMN06199224 | MiSeq 2x250bp |  |  |
| IA-P127 | SRR5140643 | SAMN06199225 | MiSeq 2x250bp |  |  |
| IA-P129 | SRR5140924 | SAMN06199227 | MiSeq 2x250bp |  |  |
| IA-P131 | SRR5140790 | SAMN06199229 | MiSeq 2x250bp |  |  |
| IA-P133 | SRR5140837 | SAMN06199230 | MiSeq 2x250bp |  |  |
| IA-P135 | SRR5140536 | SAMN06199231 | MiSeq 2x250bp |  |  |
| IA-P136 | SRR5140763 | SAMN06199232 | MiSeq 2x250bp |  |  |
| JOR-RM | SRR5140865 | SAMN06199104 | MiSeq 2x250bp |  |  |
| IA-P141 | SRR5140718 | SAMN06199236 | MiSeq 2x250bp |  |  |
| IA-P143 | SRR5140704 | SAMN06199238 | MiSeq 2x250bp |  |  |
| IA-P144 | SRR5140751 | SAMN06199239 | MiSeq 2x250bp |  |  |
| IA-P148 | SRR5140699 | SAMN06199240 | MiSeq 2x250bp |  |  |
| IA-P149 | SRR5140583 | SAMN06199241 | MiSeq 2x250bp |  |  |
| P046 | SRR5140662 | SAMN06199336 | MiSeq 2x250bp |  |  |
| P047 | SRR5140695 | SAMN06199337 | MiSeq 2x250bp |  |  |
| P048 | SRR5140621 | SAMN06199338 | MiSeq 2x250bp |  |  |
| P049 | SRR5140625 | SAMN06199339 | MiSeq 2x250bp |  |  |
| P050 | SRR5140854 | SAMN06199340 | MiSeq 2x250bp |  |  |
| P051 | SRR5140739 | SAMN06199341 | MiSeq 2x250bp |  |  |
| P052 | SRR5140533 | SAMN06199342 | MiSeq 2x250bp |  |  |
| P053 | SRR5140905 | SAMN06199343 | MiSeq 2x250bp |  |  |
| P054 | SRR5140698 | SAMN06199344 | MiSeq 2x250bp |  |  |
| P055 | SRR5140498 | SAMN06199345 | MiSeq 2x250bp |  |  |
| P056 | SRR5140908 | SAMN06199346 | MiSeq 2x250bp |  |  |
| P057 | SRR5140775 | SAMN06199347 | MiSeq 2x250bp |  |  |
| P058 | SRR5140736 | SAMN06199348 | MiSeq 2x250bp |  |  |
| P059 | SRR5140580 | SAMN06199349 | MiSeq 2x250bp |  |  |
| P060 | SRR5140910 | SAMN06199350 | MiSeq 2x250bp |  |  |
| P061 | SRR5140522 | SAMN06199351 | MiSeq 2x250bp |  |  |
| P062 | SRR5140569 | SAMN06199352 | MiSeq 2x250bp |  |  |
| P063 | SRR5140523 | SAMN06199353 | MiSeq 2x250bp |  |  |
| P064 | SRR5140530 | SAMN06199354 | MiSeq 2x250bp |  |  |
| P065 | SRR5140617 | SAMN06199355 | MiSeq 2x250bp |  |  |
| P066 | SRR5140677 | SAMN06199356 | MiSeq 2x250bp |  |  |
| P067 | SRR5140525 | SAMN06199357 | MiSeq 2x250bp |  |  |
| P068 | SRR5140688 | SAMN06199358 | MiSeq 2x250bp |  |  |
| P069 | SRR5140895 | SAMN06199359 | MiSeq 2x250bp |  |  |
| P070 | SRR5140487 | SAMN06199360 | MiSeq 2x250bp |  |  |
| P071 | SRR5140723 | SAMN06199361 | MiSeq 2x250bp |  |  |
| P072 | SRR5140502 | SAMN06199362 | MiSeq 2x250bp |  |  |
| P073 | SRR5140520 | SAMN06199363 | MiSeq 2x250bp |  |  |
| P074 | SRR5140553 | SAMN06199364 | MiSeq 2x250bp |  |  |
| P075 | SRR5140608 | SAMN06199365 | MiSeq 2x250bp |  |  |
| P076 | SRR5140618 | SAMN06199366 | MiSeq 2x250bp |  |  |
| P077 | SRR5140786 | SAMN06199367 | MiSeq 2x250bp |  |  |
| P078 | SRR5140849 | SAMN06199368 | MiSeq 2x250bp |  |  |
| P079 | SRR5140691 | SAMN06199369 | MiSeq 2x250bp |  |  |
| P080 | SRR5140707 | SAMN06199370 | MiSeq 2x250bp |  |  |
| P081 | SRR5140753 | SAMN06199371 | MiSeq 2x250bp |  |  |
| P082 | SRR5140479 | SAMN06199372 | MiSeq 2x250bp |  |  |
| P083 | SRR5140760 | SAMN06199373 | MiSeq 2x250bp |  |  |
| P084 | SRR5140620 | SAMN06199374 | MiSeq 2x250bp |  |  |
| P085 | SRR5140706 | SAMN06199375 | MiSeq 2x250bp |  |  |
| P086 | SRR5140806 | SAMN06199376 | MiSeq 2x250bp |  |  |
| P087 | SRR5140882 | SAMN06199377 | MiSeq 2x250bp |  |  |
| P088 | SRR5140902 | SAMN06199378 | MiSeq 2x250bp |  |  |
| P089 | SRR5140717 | SAMN06199379 | MiSeq 2x250bp |  |  |
| P090 | SRR5140610 | SAMN06199380 | MiSeq 2x250bp |  |  |
| P091 | SRR5140799 | SAMN06199381 | MiSeq 2x250bp |  |  |
| P092 | SRR5140869 | SAMN06199382 | MiSeq 2x250bp |  |  |
| P093 | SRR5140817 | SAMN06199383 | MiSeq 2x250bp |  |  |
| P094 | SRR5140930 | SAMN06199384 | MiSeq 2x250bp |  |  |
| P095 | SRR5140478 | SAMN06199385 | MiSeq 2x250bp |  |  |
| P096 | SRR5140767 | SAMN06199386 | MiSeq 2x250bp |  |  |
| P097 | SRR5140835 | SAMN06199387 | MiSeq 2x250bp |  |  |
| P098 | SRR5140558 | SAMN06199388 | MiSeq 2x250bp |  |  |
| P099 | SRR5140542 | SAMN06199389 | MiSeq 2x250bp |  |  |
| P100 | SRR5140843 | SAMN06199390 | MiSeq 2x250bp |  |  |
| P101 | SRR5140633 | SAMN06199391 | MiSeq 2x250bp |  |  |
| P102 | SRR5140609 | SAMN06199392 | MiSeq 2x250bp |  |  |
| P103 | SRR5140870 | SAMN06199393 | MiSeq 2x250bp |  |  |
| P104 | SRR5140694 | SAMN06199394 | MiSeq 2x250bp |  |  |
| P105 | SRR5140495 | SAMN06199395 | MiSeq 2x250bp |  |  |
| P106 | SRR5140716 | SAMN06199396 | MiSeq 2x250bp |  |  |
| P107 | SRR5140896 | SAMN06199397 | MiSeq 2x250bp |  |  |
| P108 | SRR5140856 | SAMN06199398 | MiSeq 2x250bp |  |  |
| P109 | SRR5140651 | SAMN06199399 | MiSeq 2x250bp |  |  |
| P110 | SRR5140769 | SAMN06199400 | MiSeq 2x250bp |  |  |
| P111 | SRR5140664 | SAMN06199401 | MiSeq 2x250bp |  |  |
| P112 | SRR5140747 | SAMN06199402 | MiSeq 2x250bp |  |  |
| P113 | SRR5140894 | SAMN06199403 | MiSeq 2x250bp |  |  |
| P114 | SRR5140779 | SAMN06199404 | MiSeq 2x250bp |  |  |
| P116 | SRR5140749 | SAMN06199405 | MiSeq 2x250bp |  |  |
| P117 | SRR5140537 | SAMN06199406 | MiSeq 2x250bp |  |  |
| P118 | SRR5140517 | SAMN06199407 | MiSeq 2x250bp |  |  |
| P119 | SRR5140766 | SAMN06199408 | MiSeq 2x250bp |  |  |
| P120 | SRR5140571 | SAMN06199409 | MiSeq 2x250bp |  |  |
| P122 | SRR5140588 | SAMN06199410 | MiSeq 2x250bp |  |  |
| P123 | SRR5140929 | SAMN06199411 | MiSeq 2x250bp |  |  |
| P124 | SRR5140825 | SAMN06199412 | MiSeq 2x250bp |  |  |
| P125 | SRR5140514 | SAMN06199413 | MiSeq 2x250bp |  |  |
| P126 | SRR5140710 | SAMN06199414 | MiSeq 2x250bp |  |  |
| P127 | SRR5140626 | SAMN06199415 | MiSeq 2x250bp |  |  |
| P129 | SRR5140693 | SAMN06199416 | MiSeq 2x250bp |  |  |
| P130 | SRR5140840 | SAMN06199417 | MiSeq 2x250bp |  |  |
| P131 | SRR5140541 | SAMN06199418 | MiSeq 2x250bp |  |  |
| P132 | SRR5140713 | SAMN06199419 | MiSeq 2x250bp |  |  |
| P133 | SRR5140508 | SAMN06199420 | MiSeq 2x250bp |  |  |
| P134 | SRR5140490 | SAMN06199421 | MiSeq 2x250bp |  |  |
| P135 | SRR5140863 | SAMN06199422 | MiSeq 2x250bp |  |  |
| P136 | SRR5140596 | SAMN06199423 | MiSeq 2x250bp |  |  |
| P137 | SRR5140787 | SAMN06199424 | MiSeq 2x250bp |  |  |
| IA-P001 | SRR5140702 | SAMN06199106 | MiSeq 2x250bp |  |  |
| IA-P002 | SRR5140627 | SAMN06199107 | MiSeq 2x250bp |  |  |
| IA-P004 | SRR5140731 | SAMN06199109 | MiSeq 2x250bp |  |  |
| IA-P005 | SRR5140918 | SAMN06199110 | MiSeq 2x250bp |  |  |
| IA-P006 | SRR5140689 | SAMN06199111 | MiSeq 2x250bp |  |  |
| IA-P007 | SRR5140915 | SAMN06199112 | MiSeq 2x250bp |  |  |
| IA-P008 | SRR5140492 | SAMN06199113 | MiSeq 2x250bp |  |  |
| IA-P010 | SRR5140772 | SAMN06199115 | MiSeq 2x250bp |  |  |
| IA-P011 | SRR5140534 | SAMN06199116 | MiSeq 2x250bp |  |  |
| IA-P013 | SRR5140906 | SAMN06199117 | MiSeq 2x250bp |  |  |
| IA-P015 | SRR5140532 | SAMN06199119 | MiSeq 2x250bp |  |  |
| IA-P016 | SRR5140728 | SAMN06199120 | MiSeq 2x250bp |  |  |
| IA-P018 | SRR5140674 | SAMN06199122 | MiSeq 2x250bp |  |  |
| IA-P019 | SRR5140752 | SAMN06199123 | MiSeq 2x250bp |  |  |
| IA-P020 | SRR5140494 | SAMN06199124 | MiSeq 2x250bp |  |  |
| IA-P022 | SRR5140834 | SAMN06199126 | MiSeq 2x250bp |  |  |
| IA-P023 | SRR5140497 | SAMN06199127 | MiSeq 2x250bp |  |  |
| IA-P026 | SRR5140904 | SAMN06199130 | MiSeq 2x250bp |  |  |
| IA-P027 | SRR5140579 | SAMN06199131 | MiSeq 2x250bp |  |  |
| IA-P029 | SRR5140567 | SAMN06199133 | MiSeq 2x250bp |  |  |
| IA-P030 | SRR5140512 | SAMN06199134 | MiSeq 2x250bp |  |  |
| IA-P031 | SRR5140700 | SAMN06199135 | MiSeq 2x250bp |  |  |
| IA-P032 | SRR5140540 | SAMN06199136 | MiSeq 2x250bp |  |  |
| IA-P033 | SRR5140552 | SAMN06199137 | MiSeq 2x250bp |  |  |
| IA-P034 | SRR5140712 | SAMN06199138 | MiSeq 2x250bp |  |  |
| IA-P036 | SRR5140597 | SAMN06199139 | MiSeq 2x250bp |  |  |
| IA-P037 | SRR5140690 | SAMN06199140 | MiSeq 2x250bp |  |  |
| IA-P038 | SRR5140701 | SAMN06199141 | MiSeq 2x250bp |  |  |
| IA-P040 | SRR5140655 | SAMN06199142 | MiSeq 2x250bp |  |  |
| IA-P041 | SRR5140578 | SAMN06199143 | MiSeq 2x250bp |  |  |
| IA-P042 | SRR5140500 | SAMN06199144 | MiSeq 2x250bp |  |  |
| IA-P043 | SRR5140607 | SAMN06199145 | MiSeq 2x250bp |  |  |
| IA-P044 | SRR5140531 | SAMN06199146 | MiSeq 2x250bp |  |  |
| IA-P045 | SRR5140871 | SAMN06199147 | MiSeq 2x250bp |  |  |
| IA-P046 | SRR5140680 | SAMN06199148 | MiSeq 2x250bp |  |  |
| IA-P047 | SRR5140640 | SAMN06199149 | MiSeq 2x250bp |  |  |
| IA-P048 | SRR5140647 | SAMN06199150 | MiSeq 2x250bp |  |  |
| IA-P049 | SRR5140745 | SAMN06199151 | MiSeq 2x250bp |  |  |
| IA-P050 | SRR5140636 | SAMN06199152 | MiSeq 2x250bp |  |  |
| IA-P051 | SRR5140811 | SAMN06199153 | MiSeq 2x250bp |  |  |
| IA-P052 | SRR5140488 | SAMN06199154 | MiSeq 2x250bp |  |  |
| IA-P053 | SRR5140793 | SAMN06199155 | MiSeq 2x250bp |  |  |
| IA-P054 | SRR5140683 | SAMN06199156 | MiSeq 2x250bp |  |  |
| IA-P055 | SRR5140881 | SAMN06199157 | MiSeq 2x250bp |  |  |
| IA-P056 | SRR5140818 | SAMN06199158 | MiSeq 2x250bp |  |  |
| IA-P057 | SRR5140563 | SAMN06199159 | MiSeq 2x250bp |  |  |
| IA-P058 | SRR5140848 | SAMN06199160 | MiSeq 2x250bp |  |  |
| IA-P059 | SRR5140493 | SAMN06199161 | MiSeq 2x250bp |  |  |
| IA-P060 | SRR5140510 | SAMN06199162 | MiSeq 2x250bp |  |  |
| IA-P061 | SRR5140732 | SAMN06199163 | MiSeq 2x250bp |  |  |
| IA-P064 | SRR5140844 | SAMN06199166 | MiSeq 2x250bp |  |  |
| IA-P065 | SRR5140519 | SAMN06199167 | MiSeq 2x250bp |  |  |
| IA-P066 | SRR5140780 | SAMN06199168 | MiSeq 2x250bp |  |  |
| IA-P067 | SRR5140589 | SAMN06199169 | MiSeq 2x250bp |  |  |
| IA-P068 | SRR5140792 | SAMN06199170 | MiSeq 2x250bp |  |  |
| IA-P069 | SRR5140851 | SAMN06199171 | MiSeq 2x250bp |  |  |
| IA-P071 | SRR5140711 | SAMN06199172 | MiSeq 2x250bp |  |  |
| IA-P072 | SRR5140586 | SAMN06199173 | MiSeq 2x250bp |  |  |
| IA-P073 | SRR5140889 | SAMN06199174 | MiSeq 2x250bp |  |  |
| IA-P074 | SRR5140524 | SAMN06199175 | MiSeq 2x250bp |  |  |
| IA-P075 | SRR5140516 | SAMN06199176 | MiSeq 2x250bp |  |  |
| IA-P076 | SRR5140599 | SAMN06199177 | MiSeq 2x250bp |  |  |
| IA-P077 | SRR5140622 | SAMN06199178 | MiSeq 2x250bp |  |  |
| IA-P078 | SRR5140650 | SAMN06199179 | MiSeq 2x250bp |  |  |
| IA-P079 | SRR5140768 | SAMN06199180 | MiSeq 2x250bp |  |  |
| IA-P080 | SRR5140602 | SAMN06199181 | MiSeq 2x250bp |  |  |
| IA-P081 | SRR5140807 | SAMN06199182 | MiSeq 2x250bp |  |  |
| IA-P082 | SRR5140897 | SAMN06199183 | MiSeq 2x250bp |  |  |
| IA-P085 | SRR5140669 | SAMN06199186 | MiSeq 2x250bp |  |  |
| IA-P086 | SRR5140649 | SAMN06199187 | MiSeq 2x250bp |  |  |
| IA-P087 | SRR5140819 | SAMN06199188 | MiSeq 2x250bp |  |  |
| IA-P088 | SRR5140661 | SAMN06199189 | MiSeq 2x250bp |  |  |
| IA-P089 | SRR5140538 | SAMN06199190 | MiSeq 2x250bp |  |  |
| IA-P091 | SRR5140614 | SAMN06199192 | MiSeq 2x250bp |  |  |
| IA-P092 | SRR5140675 | SAMN06199193 | MiSeq 2x250bp |  |  |
| IA-P093 | SRR5140697 | SAMN06199194 | MiSeq 2x250bp |  |  |
| IA-P095 | SRR5140788 | SAMN06199196 | MiSeq 2x250bp |  |  |
| IA-P096 | SRR5140737 | SAMN06199197 | MiSeq 2x250bp |  |  |
| IA-P098 | SRR5140505 | SAMN06199198 | MiSeq 2x250bp |  |  |
| IA-P099 | SRR5140830 | SAMN06199199 | MiSeq 2x250bp |  |  |
| IA-P100 | SRR5140735 | SAMN06199200 | MiSeq 2x250bp |  |  |
| IA-P101 | SRR5140781 | SAMN06199201 | MiSeq 2x250bp |  |  |
| IA-P155 | SRR5140926 | SAMN06199246 | MiSeq 2x250bp |  |  |
| IA-P156 | SRR5140667 | SAMN06199247 | MiSeq 2x250bp |  |  |
| IA-P157 | SRR5140744 | SAMN06199248 | MiSeq 2x250bp |  |  |
| IA-P158 | SRR5140692 | SAMN06199249 | MiSeq 2x250bp |  |  |
| IA-P159 | SRR5140722 | SAMN06199250 | MiSeq 2x250bp |  |  |
| IA-P160 | SRR5140727 | SAMN06199251 | MiSeq 2x250bp |  |  |
| IA-P161 | SRR5140638 | SAMN06199252 | MiSeq 2x250bp |  |  |
| IA-P162 | SRR5140815 | SAMN06199253 | MiSeq 2x250bp |  |  |
| IA-P163 | SRR5140652 | SAMN06199254 | MiSeq 2x250bp |  |  |
| IA-P164 | SRR5140874 | SAMN06199255 | MiSeq 2x250bp |  |  |
| IA-P165 | SRR5140544 | SAMN06199256 | MiSeq 2x250bp |  |  |
| IA-P166 | SRR5140776 | SAMN06199257 | MiSeq 2x250bp |  |  |
| IA-P167 | SRR5140598 | SAMN06199258 | MiSeq 2x250bp |  |  |
| IA-P168 | SRR5140884 | SAMN06199259 | MiSeq 2x250bp |  |  |
| IA-P169 | SRR5140665 | SAMN06199260 | MiSeq 2x250bp |  |  |
| IA-P170 | SRR5140491 | SAMN06199261 | MiSeq 2x250bp |  |  |
| IA-P171 | SRR5140709 | SAMN06199262 | MiSeq 2x250bp |  |  |
| IA-P172 | SRR5140858 | SAMN06199263 | MiSeq 2x250bp |  |  |
| IA-P173 | SRR5140572 | SAMN06199264 | MiSeq 2x250bp |  |  |
| IA-P174 | SRR5140888 | SAMN06199265 | MiSeq 2x250bp |  |  |
| IA-P175 | SRR5140773 | SAMN06199266 | MiSeq 2x250bp |  |  |
| IA-P176 | SRR5140919 | SAMN06199267 | MiSeq 2x250bp |  |  |
| IA-P177 | SRR5140916 | SAMN06199268 | MiSeq 2x250bp |  |  |
| IA-P178 | SRR5140593 | SAMN06199269 | MiSeq 2x250bp |  |  |
| IA-P179 | SRR5140877 | SAMN06199270 | MiSeq 2x250bp |  |  |
| IA-P181 | SRR5140922 | SAMN06199271 | MiSeq 2x250bp |  |  |
| IA-P182 | SRR5140822 | SAMN06199272 | MiSeq 2x250bp |  |  |
| IA-P183 | SRR5140867 | SAMN06199273 | MiSeq 2x250bp |  |  |
| IA-P184 | SRR5140826 | SAMN06199274 | MiSeq 2x250bp |  |  |
| IA-P185 | SRR5140521 | SAMN06199275 | MiSeq 2x250bp |  |  |
| IA-P186 | SRR5140584 | SAMN06199276 | MiSeq 2x250bp |  |  |
| IA-P187 | SRR5140750 | SAMN06199277 | MiSeq 2x250bp |  |  |
| IA-P188 | SRR5140687 | SAMN06199278 | MiSeq 2x250bp |  |  |
| IA-P189 | SRR5140482 | SAMN06199279 | MiSeq 2x250bp |  |  |
| IA-P190 | SRR5140630 | SAMN06199280 | MiSeq 2x250bp |  |  |
| IA-P191 | SRR5140928 | SAMN06199281 | MiSeq 2x250bp |  |  |
| IA-P192 | SRR5140561 | SAMN06199282 | MiSeq 2x250bp |  |  |
| IA-P193 | SRR5140529 | SAMN06199283 | MiSeq 2x250bp |  |  |
| IA-P194 | SRR5140845 | SAMN06199284 | MiSeq 2x250bp |  |  |
| IA-P195 | SRR5140740 | SAMN06199285 | MiSeq 2x250bp |  |  |
| IA-P196 | SRR5140515 | SAMN06199286 | MiSeq 2x250bp |  |  |
| IA-P197 | SRR5140595 | SAMN06199287 | MiSeq 2x250bp |  |  |
| IA-P198 | SRR5140539 | SAMN06199288 | MiSeq 2x250bp |  |  |
| IA-P199 | SRR5140838 | SAMN06199289 | MiSeq 2x250bp |  |  |
| IA-P200 | SRR5140898 | SAMN06199290 | MiSeq 2x250bp |  |  |
| IA-P201 | SRR5140506 | SAMN06199291 | MiSeq 2x250bp |  |  |
| P001 | SRR5140560 | SAMN06199292 | MiSeq 2x250bp |  |  |
| P002 | SRR5140615 | SAMN06199293 | MiSeq 2x250bp |  |  |
| P003 | SRR5140489 | SAMN06199294 | MiSeq 2x250bp |  |  |
| P004 | SRR5140842 | SAMN06199295 | MiSeq 2x250bp |  |  |
| P005 | SRR5140483 | SAMN06199296 | MiSeq 2x250bp |  |  |
| P006 | SRR5140503 | SAMN06199297 | MiSeq 2x250bp |  |  |
| P007 | SRR5140624 | SAMN06199298 | MiSeq 2x250bp |  |  |
| P008 | SRR5140783 | SAMN06199299 | MiSeq 2x250bp |  |  |
| P009 | SRR5140526 | SAMN06199300 | MiSeq 2x250bp |  |  |
| P010 | SRR5140883 | SAMN06199301 | MiSeq 2x250bp |  |  |
| P011 | SRR5140623 | SAMN06199302 | MiSeq 2x250bp |  |  |
| P013 | SRR5140855 | SAMN06199303 | MiSeq 2x250bp |  |  |
| P014 | SRR5140742 | SAMN06199304 | MiSeq 2x250bp |  |  |
| P015 | SRR5140527 | SAMN06199305 | MiSeq 2x250bp |  |  |
| P016 | SRR5140909 | SAMN06199306 | MiSeq 2x250bp |  |  |
| P017 | SRR5140612 | SAMN06199307 | MiSeq 2x250bp |  |  |
| P018 | SRR5140880 | SAMN06199308 | MiSeq 2x250bp |  |  |
| P019 | SRR5140887 | SAMN06199309 | MiSeq 2x250bp |  |  |
| P020 | SRR5140911 | SAMN06199310 | MiSeq 2x250bp |  |  |
| P021 | SRR5140803 | SAMN06199311 | MiSeq 2x250bp |  |  |
| P022 | SRR5140914 | SAMN06199312 | MiSeq 2x250bp |  |  |
| P023 | SRR5140657 | SAMN06199313 | MiSeq 2x250bp |  |  |
| P024 | SRR5140866 | SAMN06199314 | MiSeq 2x250bp |  |  |
| P025 | SRR5140714 | SAMN06199315 | MiSeq 2x250bp |  |  |
| P026 | SRR5140813 | SAMN06199316 | MiSeq 2x250bp |  |  |
| P027 | SRR5140616 | SAMN06199317 | MiSeq 2x250bp |  |  |
| P028 | SRR5140794 | SAMN06199318 | MiSeq 2x250bp |  |  |
| P029 | SRR5140762 | SAMN06199319 | MiSeq 2x250bp |  |  |
| P030 | SRR5140600 | SAMN06199320 | MiSeq 2x250bp |  |  |
| P031 | SRR5140682 | SAMN06199321 | MiSeq 2x250bp |  |  |
| P032 | SRR5140755 | SAMN06199322 | MiSeq 2x250bp |  |  |
| P033 | SRR5140629 | SAMN06199323 | MiSeq 2x250bp |  |  |
| P034 | SRR5140810 | SAMN06199324 | MiSeq 2x250bp |  |  |
| P035 | SRR5140501 | SAMN06199325 | MiSeq 2x250bp |  |  |
| P036 | SRR5140666 | SAMN06199326 | MiSeq 2x250bp |  |  |
| P037 | SRR5140708 | SAMN06199327 | MiSeq 2x250bp |  |  |
| P038 | SRR5140671 | SAMN06199328 | MiSeq 2x250bp |  |  |
| P039 | SRR5140507 | SAMN06199329 | MiSeq 2x250bp |  |  |
| P040 | SRR5140890 | SAMN06199330 | MiSeq 2x250bp |  |  |
| P041 | SRR5140703 | SAMN06199331 | MiSeq 2x250bp |  |  |
| P042 | SRR5140659 | SAMN06199332 | MiSeq 2x250bp |  |  |
| P043 | SRR5140603 | SAMN06199333 | MiSeq 2x250bp |  |  |
| P044 | SRR5140653 | SAMN06199334 | MiSeq 2x250bp |  |  |
| P045 | SRR5140485 | SAMN06199335 | MiSeq 2x250bp |  |  |
| P138 | SRR5140551 | SAMN06199425 | MiSeq 2x250bp |  |  |
| P140 | SRR5140852 | SAMN06199426 | MiSeq 2x250bp |  |  |
| P141 | SRR5140729 | SAMN06199427 | MiSeq 2x250bp |  |  |
| P142 | SRR5140605 | SAMN06199428 | MiSeq 2x250bp |  |  |
| P143 | SRR5140758 | SAMN06199429 | MiSeq 2x250bp |  |  |
| P144 | SRR5140568 | SAMN06199430 | MiSeq 2x250bp |  |  |
| P146 | SRR5140725 | SAMN06199431 | MiSeq 2x250bp |  |  |
| P147 | SRR5140920 | SAMN06199432 | MiSeq 2x250bp |  |  |
| P148 | SRR5140562 | SAMN06199433 | MiSeq 2x250bp |  |  |
| P149 | SRR5140668 | SAMN06199434 | MiSeq 2x250bp |  |  |
| P150 | SRR5140827 | SAMN06199435 | MiSeq 2x250bp |  |  |
| P151 | SRR5140782 | SAMN06199436 | MiSeq 2x250bp |  |  |
| P152 | SRR5140543 | SAMN06199437 | MiSeq 2x250bp |  |  |
| P157 | SRR5140724 | SAMN06199438 | MiSeq 2x250bp |  |  |
| P158 | SRR5140850 | SAMN06199439 | MiSeq 2x250bp |  |  |
