# Supplementary figures and images for: Array-based sequencing of filaggrin gene for comprehensive detection of disease-associated variants
Source: J Allergy Clin Immunol. 2018 Feb;141(2):814–6. doi: 10.1016/j.jaci.2017.10.001 (PMC5792052; doi:10.1016/j.jaci.2017.10.001)

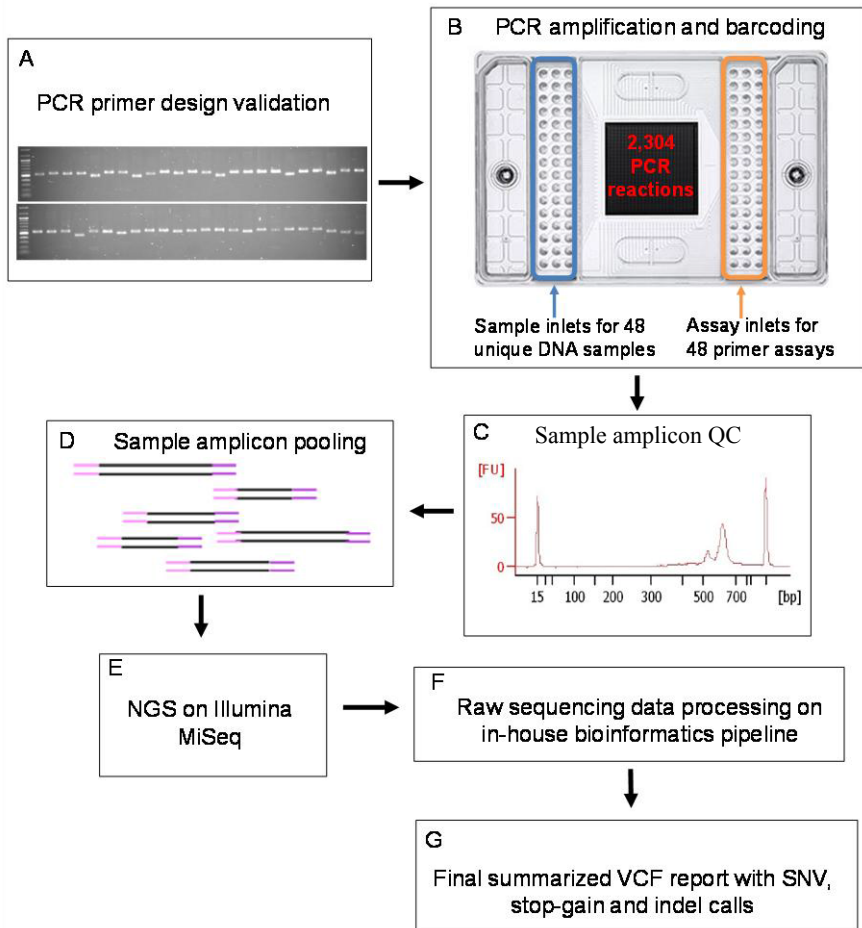

Supplement: Fig E1 [file mmc11.pdf]

A

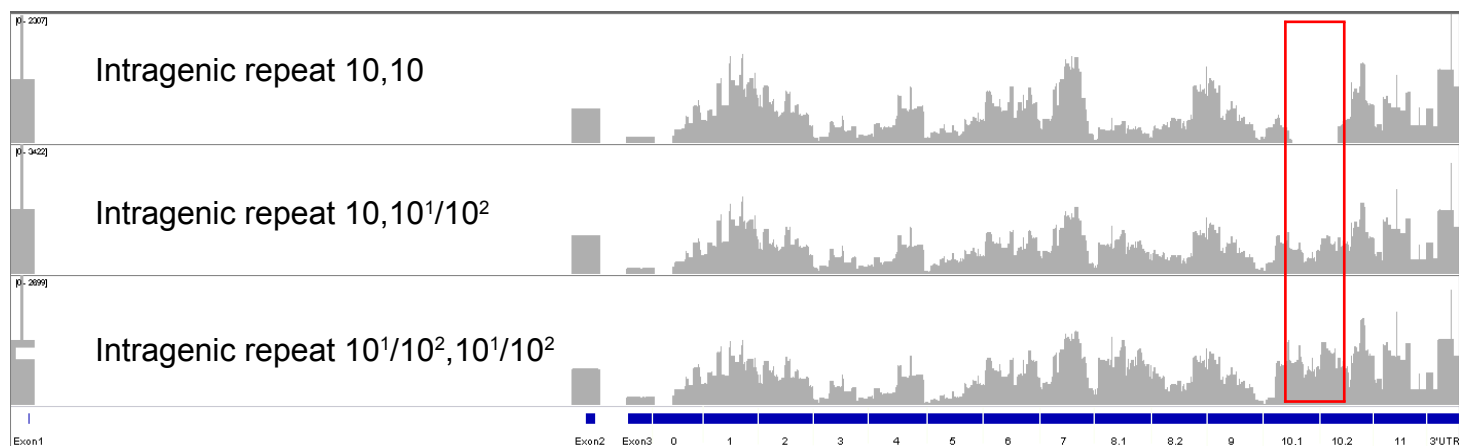

B

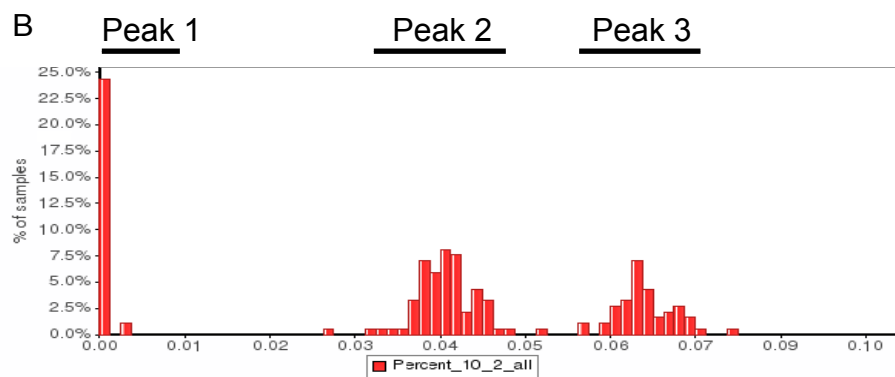

C

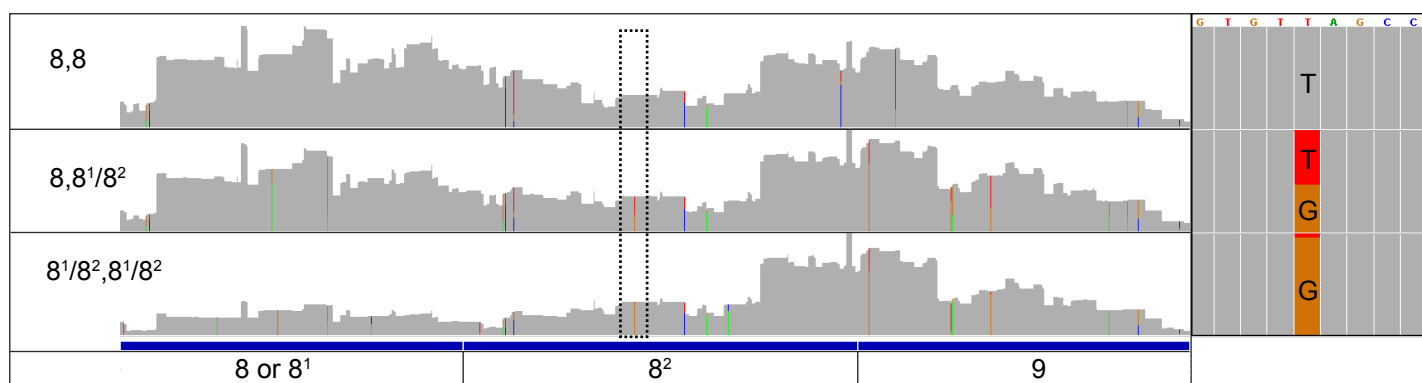

D

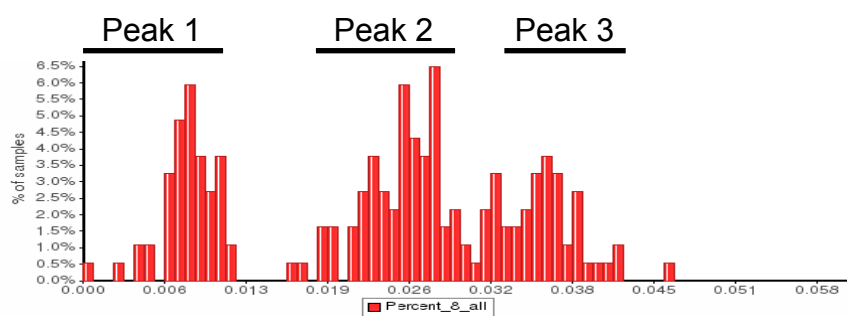

Supplement: Fig E2 [file mmc12.pdf]
