# Supplementary material for: Array-based sequencing of filaggrin gene for comprehensive detection of disease-associated variants
Source: J Allergy Clin Immunol. 2018 Feb;141(2):814–6. doi: 10.1016/j.jaci.2017.10.001 (PMC5792052; doi:10.1016/j.jaci.2017.10.001)
Supplement: Legends for Figs E1 and E2 [file mmc13.docx]

**Figure E1.** *FLG* sequencing workflow for Access Array 48.48 IFC and Illumina MiSeq.

**Figure E2. Determination of *FLG* CNV for repeat 8 and 10.** (A) The absence of a duplication of repeat 10 (repeat 10^2^) manifests as a complete drop in coverage in this region (red box). (B) Based on a defined set of coverage ratios, three distinct peaks can be generated which corresponds to each repeat 10 CNV status in a set of screened samples (Peak 1=10,10; Peak 2=10, 10^1^/10^2^; Peak 3=10^1^/10^2^, 10^1^/10^2^). (C) Repeat 8 CNV status is correlated with a single nucleotide polymorphism at position c.9645 (dotted box) of the *FLG* 12-repeat reference sequence in South-East Asian populations. (D) Three distinct peaks corresponding to repeat 8 CNV status of a sample group can be calculated based on a set of coverage ratios (Peak 1=8,8; Peak 2=8, 8^1^/8^2^ ; Peak 3=8^1^/8^2^, 8^1^/8^2^).
